# Harnessing Wind Energy for Ultraefficient Green Hydrogen Production with Tin Selenide/Tin Telluride Heterostructures

**DOI:** 10.1002/smsc.202300222

**Published:** 2024-01-14

**Authors:** Aparna Sajeev, Muthukumar Perumalsamy, Vijaykumar Elumalai, Arunprasath Sathyaseelan, Saj Anandhan Ayyappan, Monunith Anithkumar, Sang‐Jae Kim

**Affiliations:** ^1^ Nanomaterials & System Lab Major of Mechatronics Engineering Faculty of Applied Energy System Jeju National University Jeju 63243 South Korea; ^2^ Nanomaterials & System Lab Major of Mechanical System Engineering College of Engineering Jeju National University Jeju 63243 South Korea; ^3^ Research Institute of Energy New Industry (RINEI) Jeju National University Jeju 63243 South Korea

**Keywords:** anion‐exchange membrane water electrolyzers, heterostructures, hybrid water electrolyzers, tin chalcogenides

## Abstract

Industrialization of green hydrogen production through electrolyzers is hindered by cost‐effective electrocatalysts and sluggish oxygen evolution reaction (OER). Herein, a facile one‐step hydrothermal technique for the in situ growth of non‐noble tin chalcogenides and their heterostructures on nickel foam (NF) as trifunctional electrocatalysts for hydrogen evolution reaction (HER), OER, and methanol oxidation reaction (MOR) is detailed. Among them, the heterostructured SnSe/SnTe/NF outperforms all others and recently reported catalysts, boasting an impressively low potential of −0.077, 1.51, and 1.33 V versus reversible hydrogen electrode to achieve 10 mA cm^−2^ for HER, OER, and MOR. Owing to the rod‐like morphology with hetero‐phases for enhancing the performance. Furthermore, a hybrid MOR‐mediated water electrolyzer requiring only 1.49 V to achieve 10 mA cm^−2^ with value‐added formate is introduced and traditional water electrolyzer is outperformed. Additionally, a zero‐gap commercial anion‐exchange membrane water electrolyzer (AEMWE) with bifunctional SnSe/SnTe/NF electrodes is tested, successfully achieving an industrially required 1 A cm^−2^ at a low potential of 1.93 V at 70 °C. Moreover, AEMWE using a windmill is powered and H_2_ and O_2_ production with wind speed is measured. Overall, this work paves the development of unexplored tin chalcogenide heterostructure as a potent candidate for cost‐effective, energy‐efficient, and carbon‐neutral hydrogen production.

## Graphical abstract

This study unveils a one‐step hydrothermal method to synthesize tin chalcogenides and their heterostructures on nickel foam (NF). The resulting trifunctional electrocatalysts, particularly SnSe/SnTe/NF, exhibit superior performance in hydrogen evolution, oxygen evolution, and methanol oxidation reactions. The hybrid methanol oxidation reaction‐mediated water electrolyzer surpasses conventional methods, showcasing potential for industrial applications in windmill‐powered anion‐exchange membrane water electrolysis.

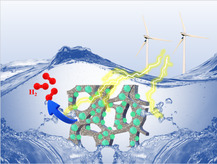

## 1 Introduction

Concern with the growing demand for energy and fossil fuel consumption develops a craving to innovate green energy technology to meet the future energy demand without deteriorating the environment.^[^ ^1^, ^2^ ^]^ Hydrogen fuel generated through electrochemical water splitting driven with green renewable energy sources of wind, solar, etc. is considered an efficient alternative clean energy source to achieve the global mission of carbon neutrality.^[^ ^3^, ^4^ ^]^ The electrochemical water splitting comprises two integral half‐cell reactions with hydrogen evolution reaction (HER) at the cathode and anodic oxygen evolution reaction (OER).^[^ ^5^ ^]^ The sluggish reaction kinetics of OER at the anode hampers the efficiency of the electrochemical water splitting, leading to excessive consumption of energy and a reduction in device efficiency.^[^ ^6^, ^7^ ^]^ To mitigate these issues, a lot of research has been carried out to improve the efficiency of OER, but no significant results have been achieved.^[^ ^8^, ^9^ ^]^ Therefore, in recent years, researchers have focused on hybridizing conventional water electrolysis with thermodynamically feasible oxidation reactions at the anodic part by eliminating the sluggish OER.^[^ ^10^, ^11^ ^]^ This hybridization allows smaller organic molecules to oxidize at lower potential with higher kinetics rather than the slower OER, resulting in low power consumption and increased hydrogen production. Organic molecules whose oxidation potential is lower than OER (1.23 V), such as alcohols,^[^ ^12^, ^13^, ^14^ ^]^ aldehydes,^[^ ^15^, ^16^ ^]^ amines,^[^ ^17^ ^]^ hydrazine,^[^ ^18^, ^19^, ^20^ ^]^ urea,^[^ ^21^ ^]^ and 5‐hydroxymethylfurfural,^[^ ^22^, ^23^, ^24^ ^]^ have been utilized in hybrid water electrolyzers (HWE) with superior results to conventional water electrolyzers. Among these organic molecules, alcohol, such as methanol, is considered more promising than others. This is attributed to its low theoretical oxidation potential of 0.03 V, simple structure, wide availability at a reasonable cost, and high selectivity in the conversion to formate, a high value‐added product.^[^ ^25^, ^26^ ^]^ The byproduct formate is a feedstock for various chemicals and can serve as a starting precursor in industries for pesticides and pharmaceuticals. Additionally, this formate has been recently utilized in green technologies such as hydrogen storage and CO_2_ capture.^[^ ^27^, ^28^ ^]^ Consequently, hybrid water electrolysis systems are becoming more prevalent. These systems use methanol electro/partial oxidation at the anode to produce useful formate instead of CO_2_ and produce green hydrogen at lower power consumption, contrasting traditional water splitting with oxygen byproducts.

Electrocatalysts play a significant role in effective H_2_ production and selective electro‐oxidation of methanol to produce formate.^[^ ^29^ ^]^ Even though the noble Pt‐based catalysts are considered benchmark electrocatalysts for HER and MOR, in the case of HWE, the noble metals lead to the total oxidation of methanol to produce CO_2_, again harming the environment.^[^ ^30^, ^31^ ^]^ In light of these challenges, researchers focus on developing cost‐effective non‐noble catalysts for HWEs with highly selective MOR without compromising hydrogen production efficiency.^[^ ^32^ ^]^ Recently, a variety of non‐noble metal sulfides,^[^ ^33^, ^34^ ^]^ selenides,^[^ ^35^ ^]^ nitrides,^[^ ^36^, ^37^ ^]^ phosphides,^[^ ^6^, ^38^ ^]^ oxides,^[^ ^39^ ^]^ borides,^[^ ^40^ ^]^ and hydroxides,^[^ ^32^, ^41^ ^]^ were reported as electrocatalysts for water electrolyzers. Among these reported electrocatalysts, metal chalcogenides are of great interest due to their cost‐effective, outstanding catalytic performance and better electrical conductivity. However, group IVA chalcogenides, particularly tin chalcogenides, have not been explored much. Furthermore, tin (Sn) is a viable alternative to transition metals due to its weaker hydrogen binding capability.^[^ ^42^ ^]^ Notably, Sn's high‐occupied d‐orbital weakens the bond strength of M*–OH, enhancing the OER process.^[^ ^43^ ^]^ Recent reports highlight the outstanding performance of tin‐based electrocatalysts, including 3D Sn/NF, for HER, OER, and MOR applications.^[^ ^44^, ^45^ ^]^ Earth‐abundant materials, including Sn‐based chalcogenides, are emerging as promising catalysts for water splitting. Through density functional theory (DFT) calculations on diverse metal chalcogenides, Qian Wu et al. discovered that tin monochalcogenides exhibit a hydrogen adsorption‐free energy (ΔGH*) close to zero. Notably, these tin monochalcogenides surpass the HER performance of the noble metal Pt electrocatalyst, which is attributed to the presence of metal vacancies.^[^ ^46^, ^47^, ^48^ ^]^ Experimentally, some researchers proved that tin chalcogenides, such as tin selenides (SnSe, SnSe_2_) and tin sulfides (SnS_2_, SnS), have been found to show promising activity and stability for electrochemical water splitting and the tin alloys are reported to improve the stability and performance toward MOR.^[^ ^49^, ^50^, ^51^ ^]^ This high activity is due to their unique electronic structure with suitable active sites, tunable morphological features with structural merits, and higher electrical conductivity, which are suitable features for the water‐splitting reaction.^[^ ^52^ ^]^ Among tin/other metallic chalcogenides, sulfur and selenides are more explored compared to tellurides. In view of tellurides, which are more superconducting and metallic in nature compared to selenides and sulfides and capable of forming chalcogenides with tin, it is better to investigate the electrocatalytic properties of metal tellurides. It is well documented that creating heterostructured chalcogenides is more interesting than single‐phase chalcogenides. Compared to the individual components, the heterostructure facilitates the charge transfer and adsorption sites.^[^ ^53^ ^]^ Also, the bandgap difference in the heterostructure will create an internal electric field due to more efficient charge transfer.^[^ ^54^ ^]^ The formed interfaces at the heterostructure can facilitate charge transfer between the two components, enhancing intrinsic reactivity, tuning binding affinity to reaction intermediates, and ultimately enhancing reaction kinetics synergistically.^[^ ^55^ ^]^ Further, many researchers, through DFT, proved that compared to single‐phase components, the heterostructures exhibited smaller hydrogen adsorption free energy (ΔGH*) due to the higher rate of H_2_O dissociation due to interfacial charge transfer and increased density of states near the Fermi level.^[^ ^45^, ^56^, ^57^ ^]^

Inspired by the above features, herein, for the first time, we develop a non‐noble tin chalcogenide heterostructure on nickel foam (NF) by a facile and green one‐step hydrothermal process. The synthesized catalysts exhibit better electrocatalytic performance toward HER, OER, and MOR. Notably, the synthesized tin chalcogenide heterostructure of SnSe/SnTe/NF performance was superior due to the rod‐like morphology with increased accessibility/exposure of active sites and heterophase tuning the unique electronic properties. Further, we analyzed an energy‐saving HWE (methanol mediated) with the SnSe/SnTe/NF as a bifunctional catalyst to produce hydrogen, outperforming the traditional water electrolyzer and producing hydrogen and value‐added formate as a byproduct. We also fabricated a commercial zero‐gap anion exchange membrane water electrolyzer (AEMWE) and integrated it with a windmill for real‐world application with the SnSe/SnTe/NF as a bifunctional catalyst. Utilizing a one‐step hydrothermal technique for advanced interface engineering in constructing non‐noble metal electrocatalysts, this approach addresses the gap in Sn‐based materials for comprehensive water splitting.

## 2 Results and Discussion

### 2.1 Morphological and Structural Analysis

**Figure 1:**
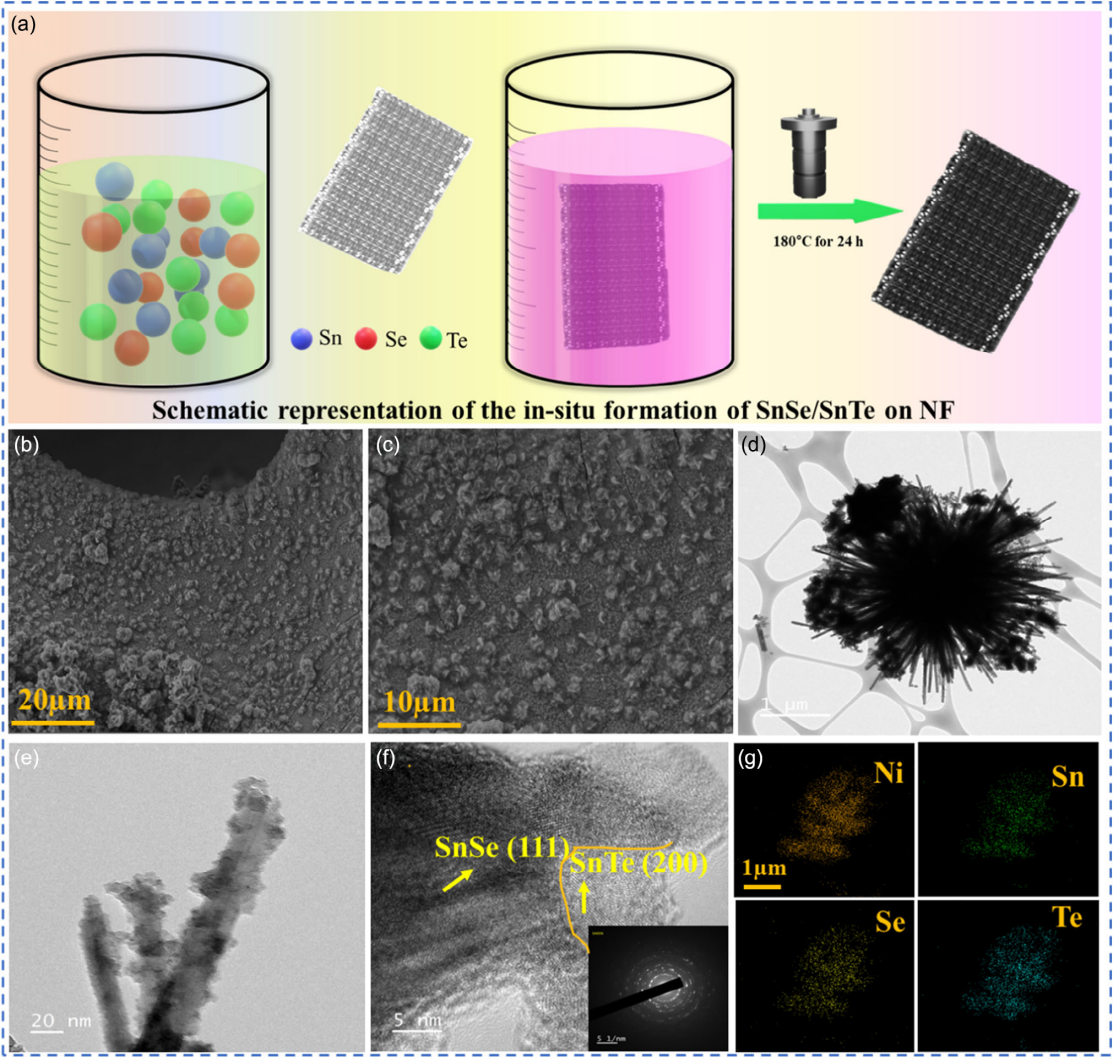
a) Schematic representation of the in situ formation of SnSe/SnTe on NF using a facile hydrothermal technique and morphological characterizations of SnSe/SnTe nanorod. b,c) FESEM images of SnSe/SnTe/NF and d–f) HRTEM images at different magnification and inset in (f) depicts the SAED pattern of SnSe/SnTe heterostructure with polycrystalline nature. g) HRTEM EDS elemental mapping of SnSe/SnTe.

**Figure 2:**
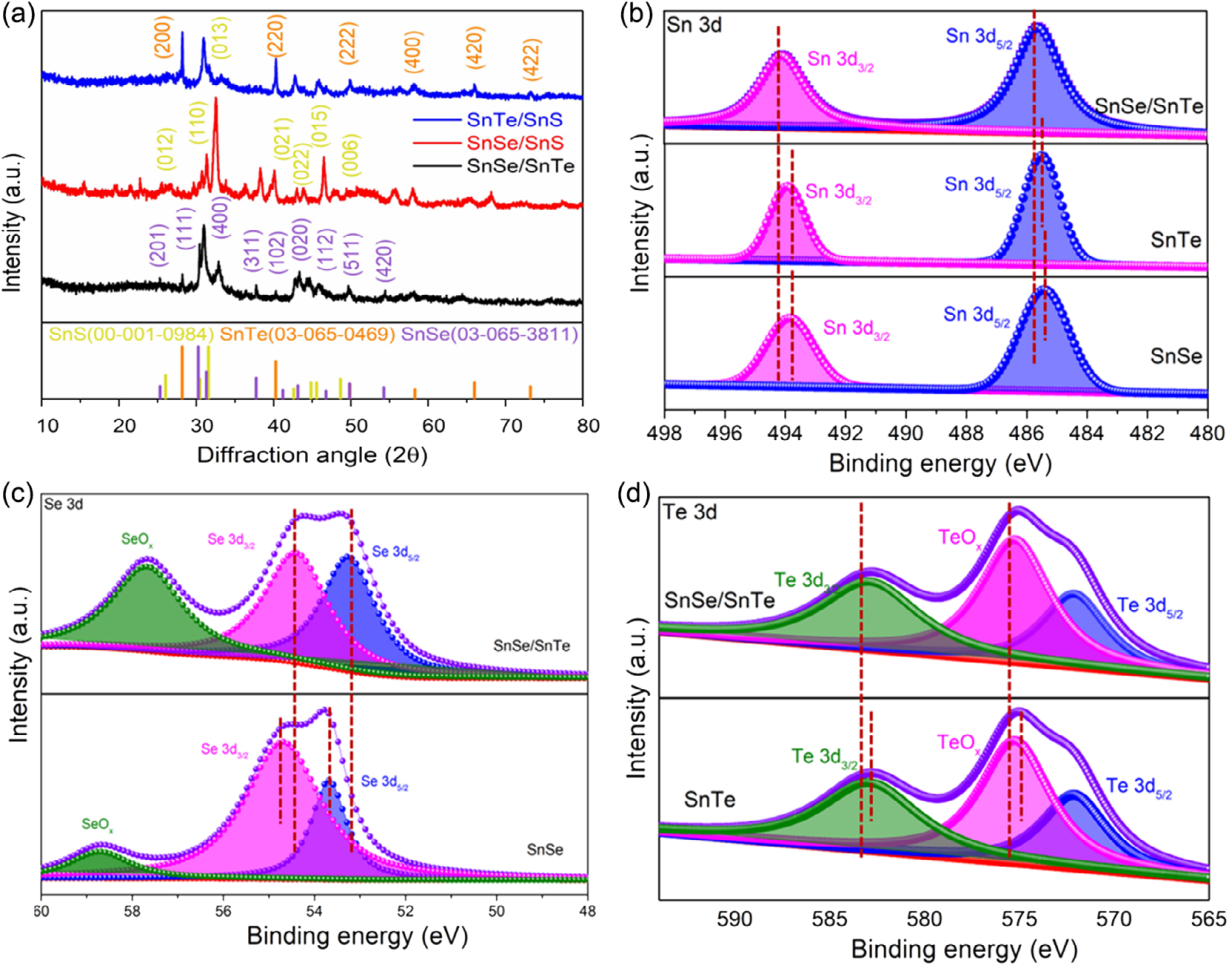
Physiochemical characterization of SnSe/SnTe/NF nanorod heterostructure. a) XRD analysis of SnSe/SnTe, SnSe/SnS, and SnS/SnTe heterostructured powder sample. XPS core level; spectrum of b) Sn 3*d*, c) Se 3*d*, and d) Te 3d of SnSe/SnTe/NF.

The heterostructure formation of SnSe/SnTe, SnSe/SnS, and SnS/SnTe was carried out in a single‐step facile hydrothermal process. In SnSe/SnTe/NF, simultaneous selenization and tellurization were carried out with the in situ formation of rich‐phase interfaces. The schematic representation for SnSe/SnTe/NF synthesis is shown in **Figure** **1**a. The synthesized SnSe/SnTe/NF heterostructure acts as a binder‐free catalyst layer, liquid/gas diffusion layer, and current collector. The surface morphology of SnSe/SnTe on NF with different magnifications is shown in Figure 1b,c, which manifests the even distribution of samples all over the surface of Ni foam. Similarly, the field‐emission scanning electron microscope (FESEM)–energy‐dispersive X‐ray spectroscopy (EDX) analysis of SnSe/SnS/NF, SnS/SnTe/NF, SnSe/NF, SnTe/NF, and SnS/NF is shown in Figure S1–S3, Supporting Information. The EDX analysis confirms the presence of respective elements and uniform distribution on the NF. The transmission electron microscopy (TEM) images with different magnifications of SnSe/SnTe are displayed in Figure 1d–f, exhibiting the nanorod‐like morphology and the aggregation of nanorod‐like structures to form flower‐like morphologies. Further, the TEM–EDX elemental mapping of Ni, Sn, and Te is depicted in Figure 1g, which further demonstrates that the mixed phases of SnSe and SnTe are homogeneously distributed on the nanorod surface, and the resultant heterointerphases are beneficial for the electrochemical water splitting. In addition, the high‐resolution TEM (HRTEM) analysis with the selected area electron diffraction (SAED) pattern of SnSe/SnTe is depicted in Figure 1f. The SAED pattern displayed the polycrystalline nature of the SnSe/SnTe. It also shows a d‐spacing value of 0.28 nm and 0.31 nm corresponding to SnSe and SnTe, respectively.^[^ ^58^ ^]^ Furthermore, XRD spectroscopy is used to analyze the chemical, crystal structure of the synthesized SnSe/SnTe, SnSe‐SnS, and SnS‐SnTe in powder form is shown in **Figure** **2**a, and the XRD pattern of SnSe/SnTe/NF, SnSe/SnS/NF, SnS/SnTe/NF, SnSe/NF, SnS/NF, and SnTe/NF is shown in Figure S4, Supporting Information. The prominent XRD peaks of SnSe‐SnTe/NF at 28.23 and 40.27 correspond to the (200) and (220) planes of SnTe, 30.36, 37.78, 43.28, and 49.78 correspond to the (111), (311), (020), and (511) plane of SnSe. Noticeably, the remaining less intense peaks correspond to the hexagonal structure of NiSe and NiTe due to the reaction of NF substrate, similar to the previous report.^[^ ^46^ ^]^ Similarly, for SnSe/SnS and SnS/SnTe, the peaks corresponding to the SnSe, SnS, NiSe, NiS and SnS, SnTe, and NiS and NiTe were seen, respectively. The XRD results suggest the successful formation of the phase‐rich heterostructures of all the prepared catalysts. For comparison, XRD of SnSe, SnTe, and SnS is also shown, which matches with the diffraction pattern of reported literature and ICSD (03‐065‐3811), (03‐065‐0469), and (00‐001‐0984), respectively, conforming the orthorhombic structure. The XPS technique was employed to analyze the valence state and surface chemical composition of SnSe/SnTe/NF. The presence of Sn, Se, Te, and Ni is conformed from the survey spectrum, indicating the successful formation of heterostructured SnSe/SnTe/NF, as depicted in Figure S5, Supporting Information. The deconvoluted high‐resolution XPS core‐level spectrum of Sn 3*d* is shown in Figure 2b, which explains two significant peaks at 485.6 and 494.11 eV corresponding to Sn 3*d*_5/2_ and Sn 3*d*_3/2_, indicating the existence of Sn^2+^ in SnSe and SnTe. Compared to pure SnSe, the two peaks of Sn 3*d* spectra are positively shifted in SnSe/SnTe/NF, suggesting the strong electronic interaction between the SnSe and SnTe.^[^ ^45^, ^59^, ^60^ ^]^ The core‐level spectrum of Se is deconvoluted into doublet peaks at binding energies of 53.27 and 54.45 eV attributed to the Se3*d*_5/2_ and Se 3*d*_3/2_, an additional peak at 57.71 is observed, which is attributed to the SeO_*x*_ species due to unavoidable surface oxidation (Figure 2c).^[^ ^61^ ^]^ Compared to SnSe, the Se 3*d* peaks are negatively shifted in SnSe/SnTe, suggesting that extra charges in Se imply an increased capability to adsorb H* and OH*intermediates.^[^ ^62^ ^]^ Figure 2d exhibits the deconvoluted XPS spectra of Te 3*d*, which depict the peaks at binding energies of 582.7 and 572.3 eV corresponding to Te 3*d*_3/2_ and Te 3*d*_5/2_.^[^ ^63^ ^]^ The peak at a binding energy of 575.33 eV corresponds to the partial oxidation of Te on the surface. The positive shift of Te 3*d* in SnSe/SnTe/NF relative to that of pure SnTe suggests electron depletion. Crucially, the binding energies of the Sn 3*d* and Te 3*d* peaks in SnSe/SnTe/NF are all positively shifted as compared to pure SnTe, indicating that SnTe's electrons were depleted following the SnSe alteration. The intriguing occurrence can be clarified by the impact of charge transfer from tin and tellurium to selenium, which further modifies the SnSe/SnTe/NF electronic structure. The outcomes suggest the successful creation of heterostructures involving SnSe/SnTe/NF, showcasing effective interactions that modulate the electronic structure. This, in turn, enhances the adsorption of hydrogen and oxygen intermediates onto the electrocatalyst's surface.

Moreover, we further analyzed the Brunauer–Emmett–Teller (BET) specific surface area (*S*_BET_) using N_2_ adsorption/desorption isotherms, as illustrated in Figure S17, Supporting Information. The results indicate that all the catalysts display a Type IV isotherm.^[^ ^31^ ^]^ Specifically, SnSe/SnTe exhibits a significantly increased surface area (65.6 m^2^ g^−1^) compared to SnSe/SnS (45.8 m^2^ g^−1^), SnS/SnTe/NF (45.2 m^2^ g^−1^), SnSe/NF (36.4 m^2^ g^−1^), SnS/NF (22.2 m^2^ g^−1^), and SnTe/NF (17.0 m^2^ g^−1^).^[^ ^64^ ^]^ The results collectively affirm the successful formation of phase‐rich interfaces in these heterostructures, achieved through a straightforward one‐pot hydrothermal technique. The resulting heterointerphases are poised to play a crucial role in enhancing the trifunctional performances of the HER, OER, and MOR.

### 2.2 Electrocatalytic Performance

#### 2.2.1 Hydrogen Evolution Reaction

**Figure 3:**
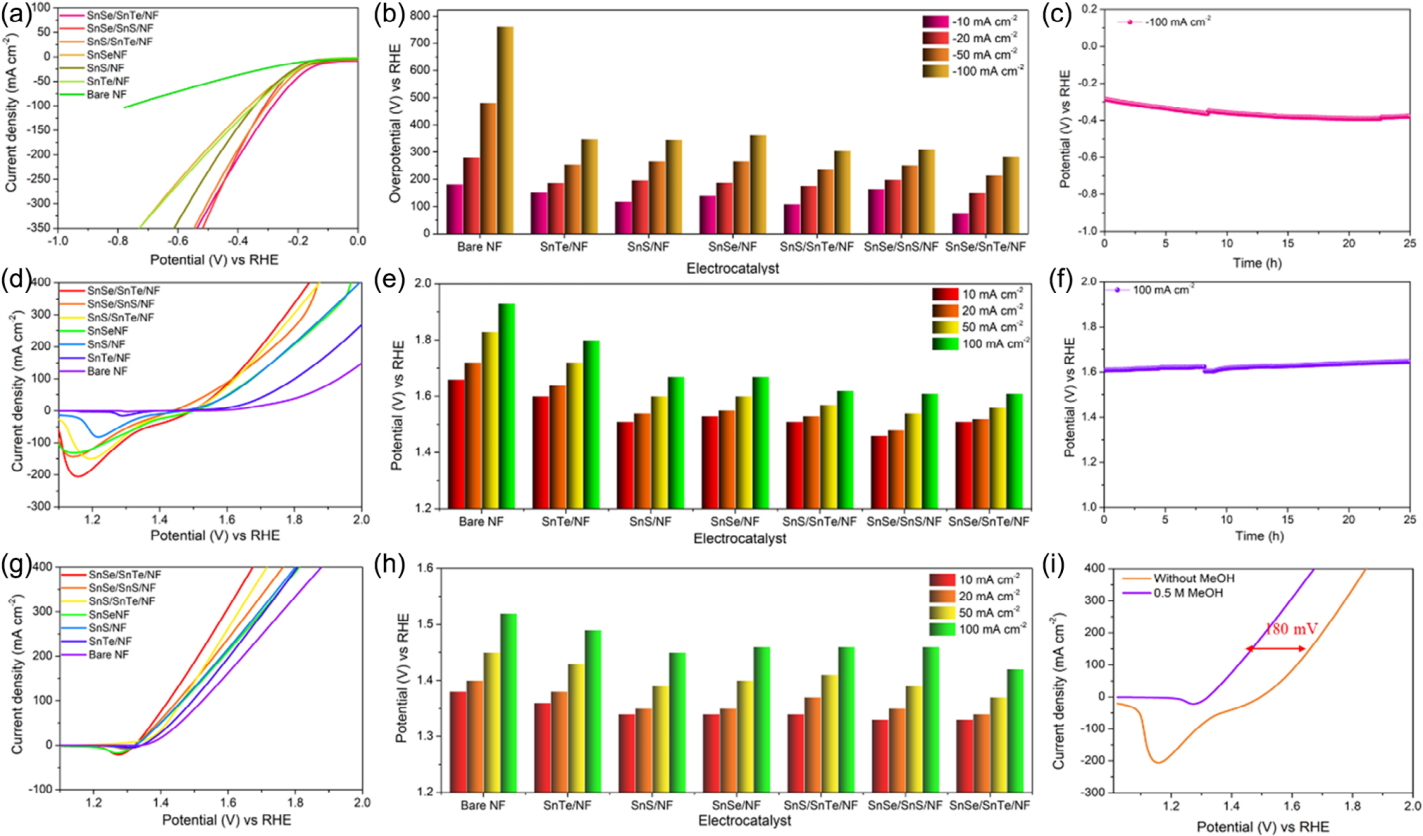
Three‐electrode electrochemical measurements of synthesized SnSe/SnTe/NF, SnSe/SnS, and SnS/SnTe, SnSe, SnS, SnTe electrocatalysts toward HER, OER, and MOR. a) HER profiles of all electrodes in 1.0 m KOH at a scan rate of 5 mV s^−1^ (without *iR* correction). b) The overpotential comparison of all the catalysts at different current densities. c) Stability measurement of SnSe/SnTe/NF up to 25 h for HER by applying a constant current of −100 mA cm^−2^. d) OER performance of SnSe/SnTe/NF, SnSe/SnS, and SnS/SnTe, SnSe, SnS, SnTe, and bare NF electrodes in 1.0 m KOH at a scan rate of 5 mV s^−1^ (without *iR* correction). e) The overpotential comparison of all the catalysts at different current densities. f) Stability measurement of SnSe/SnTe/NF up to 25 h for OER by applying a constant current of 100 mA cm^−2^. g) MOR performance of SnSe/SnTe/NF, SnSe/SnS and SnS/SnTe andSnSe, SnS, SnTe, and bare NF electrodes in 1.0 m KOH with 0.5 m CH_3_OH at a scan rate of 5 mV s^−1^. h) The MOR potential comparison of all the catalysts at different current densities. i) LSV polarization curve of SnSe/SnTe/NF with and without CH_3_OH.

Inspired by the changes in the electronic structure of SnSe/SnTe/NF heterostructures, the electrochemical activity of all the prepared catalysts toward HER is investigated using a three‐electrode setup. The HER performance of all the prepared catalysts is depicted in **Figure** **3**a. The LSV curve shows that the SnSe/SnTe/NF exhibits better catalytic performance compared to other prepared catalysts, with a less overpotential of 77 mV to reach a current density of 10 mA cm^−2^, whereas the SnSe/SnS/NF, SnS/SnTe/NF, SnSe/NF, SnS/NF, SnTe/NF, and bare NF require 165, 109, 140, 118, 153, and 182 mV versus RHE. The remarkable performance of SnSe/SnTe/NF is due to the nanorod‐like morphology of the catalysts, which provides a high surface area with accessible active sites, as evidenced from BET. The performance of SnSe/SnTe/NF is higher or in par with recent literature, as shown in Table S1, Supporting Information. A comparative chart of the overpotential of all the catalysts at various current densities is exhibited in Figure 3b, which illustrates that SnSe/SnS/NF requires only smaller overpotential of 150, 215, and 283 mV to achieve current densities of 20, 50, and 100 mA cm^−2^ respectively. To analyze the kinetics rate change of the catalysts toward HER further, we conducted the Tafel slope, as depicted in Figure S7, Supporting Information. The results reveal that SnSe/SnTe/NF exhibited a smaller slope value of 142 mV dec^−1^ compared to SnSe/SnS/NF (158 mV dec^−1^), SnS/SnTe/NF (165 mV dec^−1^), SnSe/NF (191 mV dec^−1^), SnS/NF (205 mV dec^−1^), and SnTe/NF (178 mV dec^−1^). This suggests superior kinetics for HER, with the rate‐limiting step being the Volmer–Heyrovsky reaction. In order to further explore the intrinsic electrocatalytic activity of catalysts, EIS measurements were executed by using a bias potential of −0.227 V versus RHE (Figure S12a, Supporting Information). It clearly depicts that SnSe/SnTe/NF has a less *R*_s_ value of 0.50 Ω compared to other catalysts and a smaller radius of arc, which explains the more effective charge transfer and increased kinetics of the catalysts. This is accomplished by reducing electron–hole recombination and increasing the number of electron trapping sites, which offer increased surface area and accelerate electron transfer.^[^ ^65^ ^]^ Moreover, the increased charge transfer can be attributed to the internal electric field due to the heterostructure.^[^ ^45^ ^]^ Moreover, for an in‐depth assessment of the essential factors influencing practicability, electrochemical stability, and rate capability of the SnSe/SnTe/NF catalysts in the HER context, we conducted chronopotentiometry. This involved applying a constant current density of −100 mA cm^−2^ for a duration of 25 h and subsequently subjecting the catalysts to a multistep current profile, with increments of 10, 20, 30, 40, and 50 mA cm^−2^, followed by a return to 10 mA cm^−2^ for an additional 25 h. The chronopotentiometric curve shows that almost 93% of potential was retained after 25 h (Figure 3c). In addition to the stability, the rate performance (Figure S8a, Supporting Information) of the catalyst was analyzed using current density from lower to higher and again to lower range, surprisingly the SnSe/SnTe/NF demonstrated improved rate performance at all current densities, indicating its longevity. Moreover, Figure S8b, Supporting Information, presents the LSV curve following 50 h of durability tests. The graph illustrates a mere 7 mV change in the LSV curve, indicating the catalysts’ remarkable stability in achieving a current density of 100 mA cm^−2^ during the HER process.

#### 2.2.2 Oxygen Evolution Reaction

The LSV technique was used to investigate the OER measurements of various configurations, including SnSe/SnTe/NF, SnSe/SnS/NF, SnS/SnTe/NF, SnSe/NF, SnS/NF, SnTe/NF, and bare NF, as illustrated in Figure 3d. The results reveal that SnSe/SnTe/NF demonstrates outstanding OER performance, achieving a potential of 1.51 V versus RHE, surpassing SnSe/SnS/NF, SnS/SnTe/NF, SnSe/NF, SnS/NF, SnTe/NF, and bare NF, to attain a current density of 10 mA cm^−2^. Based on the evaluation metrics, SnSe/SnTe requires only a less overpotential of 280 mV, and the performance was superior to most of the recently reported catalysts, as shown in Table S2, Supporting Information. A comparison of the potential required to drive OER with a current density of 10, 20, 50, and 100 mA cm^−2^ for all the catalysts is shown in Figure 3e, which follows a similar trend. Further, to understand the inherent kinetics of OER, Tafel slope analysis was carried out and is shown in Figure S9, Supporting Information. The Tafel slope value of SnSe/SnTe/NF is 86 mV dec^−1^, lower compared to SnSe/SnS/NF (129 mV dec^−1^), SnS/SnTe/NF (94 mV dec^−1^), SnSe/NF (119 mV dec^−1^), SnS/NF(143 mV dec^−1^), and SnTe/NF(169 mV dec^−1^). The Tafel values indicate that the OER of these catalysts follows the Krasil`shcikov pathway, that is, the OER mechanism occurs at the surface of the catalysts, as explained in the mechanism given in Equation (S6–S9), Supporting Information. The evident higher catalytic activity of heterostructures in contrast to homogeneously structured SnSe, SnTe, and SnS toward OER is attributed to improved carrier transport, enhanced electron transfer due to the internal electric field, and greater accessibility to active sites. This distinction highlights the enhanced performance of the composite materials over their individual counterparts. Out of the three heterostructures (SnSe/SnTe/NF, SnSe/SnS/NF, and SnS/SnTe/NF), SnSe/SnTe/NF outperforms better than the other two because of the rod‐like morphology, which provides a high aspect ratio, thereby increasing surface area as evidenced from BET, internal electric field due to the formation of bandgap difference, and accessible active sites. In addition, EIS analysis was carried out to understand the reaction kinetics of the catalysts toward OER using a bias potential of 1.57 V versus RHE (Figure S12b, Supporting Information). In this, SnSe/SnTe/NF exhibits a lower diameter of arc, indicating a higher rate of charge transfer between the interface of electrode and electrolyte due to the electric field and charge transfer between the heterostructures. Furthermore, we analyzed the durability of the catalysts using chronopotentiometry and multicurrent analysis. The chronopotentiometry analysis in Figure 3f exhibits a negotiable change in potential after 25 h of applying a constant current density of 100 mA cm^−2^. Moreover, as shown in Figure S10a, Supporting Information, multiple current densities varying from 10 to 50 mA cm^−2^ were applied for 25 h to investigate its rate performance toward OER. From lower to higher, it exhibits an excellent rate performance with a change in the potential for different currents and almost constant for the same current. The LSV analysis before and after 50 h of stability is given in Figure S10b, Supporting Information, which shows only a negligible change of 0.03 V in the curve @100 mA cm^−2^, depicting the stability of catalysts. These studies suggest the catalyst's excellent stability and superior performance toward OER.

#### 2.2.3 Methanol Oxidation Reaction (MOR)

The MOR measurements of all the prepared catalysts were analyzed in the three‐electrode setup similar to HER and OER with an electrolyte containing 0.5 m CH_3_OH. Figure 3g shows the polarization curve of all catalysts in 1 m KOH containing 0.5 m CH_3_OH. Figure 3g depicts that SnSe/SnTe/NF requires only a significantly less potential of 1.42 V versus RHE to reach a current density of 100 mA cm^−2^ compared to SnSe/SnS/NF(1.44 V vs RHE), SnS/SnTe/NF(1.46 V vs RHE), SnSe/NF(1.46 V vs RHE), SnS/NF (1.46 V vs RHE), SnTe/NF (1.49 V vs RHE), and bare NF(1.52 V vs RHE). Figure 3i presents the LSV curve for SnSe/SnTe/NF, both with and without CH3OH. Notably, the potential required to achieve a current density of 10 mA cm^−2^ in the MOR is only 1.33 V versus RHE, showcasing a reduction of 0.18 V compared to the anodic OER process. This observation underscores the heightened responsiveness of the catalysts to MOR. This superior performance can be ascribed to the heterostructure interfaces inducing electronic modulation and the synergetic effect, potentially promoting MOR through enhanced methanol adsorption and active dissociation of methanol. Apart from this, the tin monochalcogenide with a metal vacancy and the interface between the NF support and the metal heterostructure may also provide electron transfer that is beneficial for MOR. In addition, the Sn element will influence the MOR by lowering the oxidation potential and further promoting oxidation by OH_ads_.^[^ ^66^ ^]^ Furthermore, the LSV curve is used to extrapolate the Tafel slope to analyze the kinetics of catalysts toward MOR, which shows the smaller Tafel slope value of SnSe/SnTe/NF (70 mV dec^−1^) compared to SnSe/SnS/NF(91 mV dec^−1^) and SnS/SnTe/NF (106 mV dec^−1^), SnSe/NF (101 mV dec^−1^), SnS/NF (96 mV dec^−1^), SnTe/NF (106 mV dec^−1^), and bare NF (122 mV dec^−1^) (Figure S11, Supporting Information). A comparison of the potential requisited to achieve a current density of 10, 20, 50, and 100 mA cm^−2^ is shown in Figure 3h, clearly demonstrating the less potential required for SnSe/SnTe/NF to reach the particular current density compared to others. Furthermore, we conducted EIS for a more in‐depth analysis of the catalyst kinetics. The results reveal a higher charge transfer rate, indicating an expanded electrode–electrolyte interface and increased adsorption–desorption on the surface of the SnSe/SnTe/NF catalysts. This finding aligns with the previously discussed superior performance and kinetics, providing additional evidence for the enhanced catalytic properties of the SnSe/SnTe/NF catalysts. Apart from the performance and kinetics, it is essential to realize the durability of the catalyst toward MOR. The durability of the catalyst toward MOR was analyzed in electrolytes containing 1 m KOH and 0.5 m CH_3_OH using chronopotentiometric analysis at 100 mA cm^−2^ for 25 h (Figure S14a, Supporting Information). A small change was observed here; it can be attributed to the continuous oxidation of methanol (irreversible process), evidenced by the LSV before and after stability (Figure S14b, Supporting Information).

To further evaluate the electrochemical active surface area (ECSA) of all the prepared electrocatalysts, cyclic voltammetry tests were carried out in the non‐Faradaic region (from 0.64 to 0.74 V vs RHE) with different scan rates (20, 40, 60, 80, and 100 mV s^−1^) in 1 M KOH electrolyte.^[^ ^64^ ^]^ As shown in Figure S18, Supporting Information, SnSe/SnTe/NF exhibits largest double‐layer capacitance (*C*_dl_) value of 11.2 mF cm^−2^ compared to SnSe/SnS/NF (7.8 mF cm^−2^), SnS/SnTe/NF (3.7 mF cm^−2^), SnSe/NF (2.1 mF cm^−2^), SnS/NF (1.9 mF cm^−2^), and SnTe/NF (1.6 mF cm^−2^). The substantial double‐layer capacitance (*C*_dl_) provides evidence for the abundant active sites and enhanced intrinsic activity of SnSe/SnTe/NF, contributing to its outstanding performance in the HER, OER, and MOR. The ECSA of the catalyst can be determined by dividing the double‐layer capacitance (*C*_dl_) by the specific capacitance (*C*_s_ = 40 μF cm^−2^). The calculated ECSA value of SnSe/SnTe/NF is 280 cm^2^, much higher than that of others.

The overall electrochemical performance of SnSe/SnTe/NF toward HER, OER, and MOR underscores its potential as a promising electrocatalyst. With superior performance and stability, these characteristics are indispensable for realizing energy‐efficient hydrogen production in real‐time applications. The excellent performance of heterostructure over the single components for HER, OER, and MOR is attributed to the following reasons: 1) direct integration of heterostructure on the NF, resulting in efficient electron transfer kinetics; 2) elimination of insulating binders in electrode fabrication; 3) presence of hierarchical structures on the NF as evident from TEM analysis; 4) heterostructures with effective interactions between the single phases, thereby modulating the electronic structure (as evident from XPS) and consequently improving the way hydrogen and oxygen intermediates adsorbing to electrocatalysts’ surface; and 5) combining the single components with different bandgaps in the heterostructure will create an internal electric field, leading to enhanced charge transfer. Compared to other heterostructures such as SnSe/SnS/NF and SnS/SnTe/NF, the unique morphology of SnSe/SnTe with the rod‐like structure enhances the surface area and provides more active sites. The larger BET surface area (65.6 m^2^ g^−1^) and ECSA values of SnS/SnTe/NF (280 cm^2^) also enhance performance. The higher electrical conductivity possessed by the SnSe/SnTe/NF leads to a better charge transfer, as evident from the smaller *R*_s_ and *R*_ct_ values obtained from the EIS by applying the specific potential of HER, OER, and MOR.

#### 2.2.4 Methanol‐Mediated HWE

**Figure 4:**
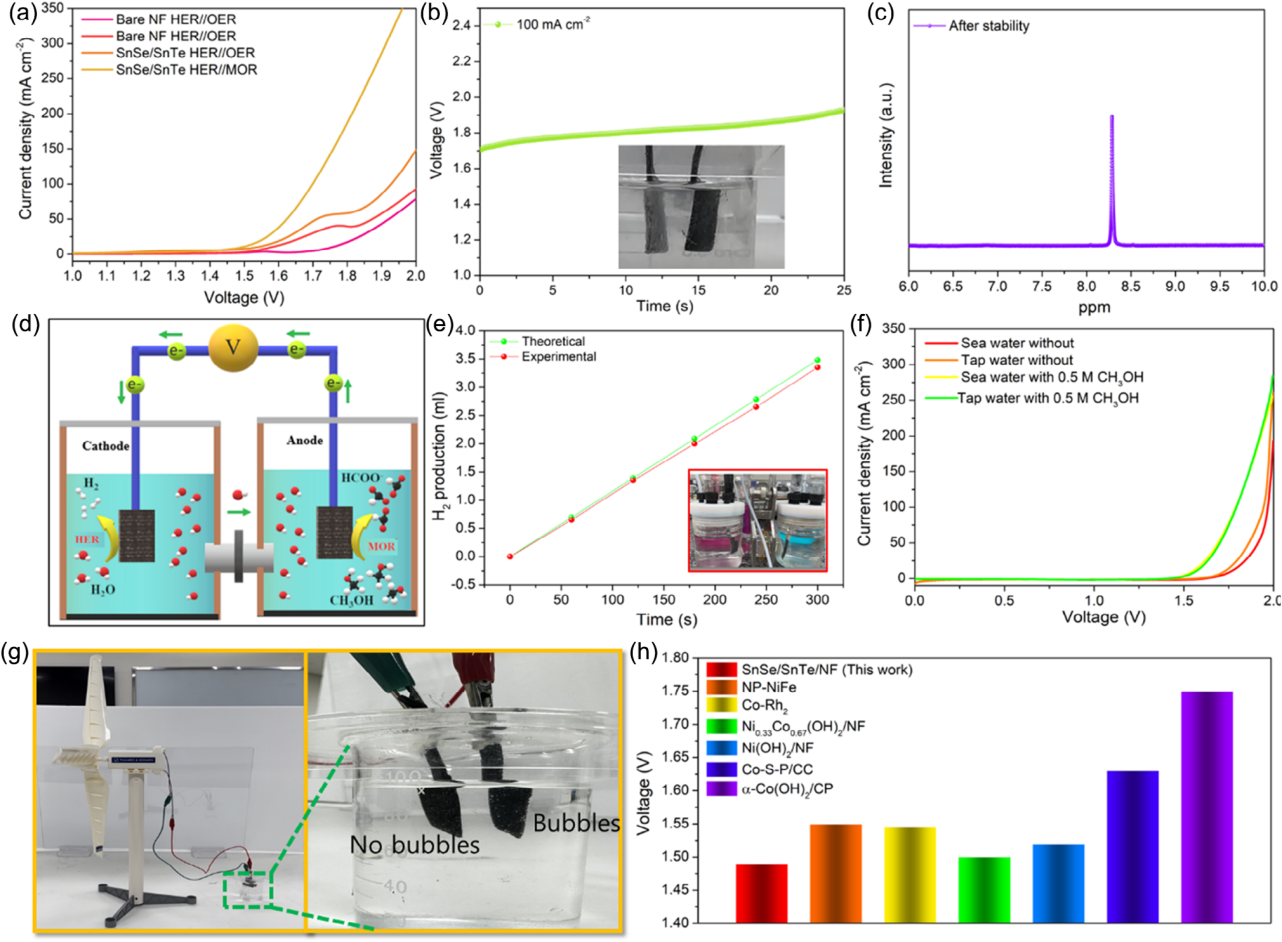
Traditional and HWE performance of SnSe/SnTe/NF//SnSe/SnTe/NF electrolyzer. a) LSV polarization curve of SnSe/SnTe/NF and bare NF as bifunctional electrocatalyst 1.0 m KOH electrolyte with and without 0.5 m CH_3_OH. b) Stability performance of HWE with SnSe/SnTe/NF as bifunctional electrode at 100 mA cm^−2^. c) ^1^H NMR measurement of formate produced during the stability test. d) Schematic representation of H‐type hybrid membrane water electrolyzer used to measure hydrogen produced. e) Theoretical and experimental H_2_ gas produced at various time intervals. f) LSV curve of SnSe/SnTe/NF//SnSe/SnTe/NF taken in tap water and sea water with and without 0.5 m CH_3_OH. g) Practical demonstration of windmill‐driven HWE. h) Comparison of performance of SnSe/SnTe/NF hybrid electrolyzer with recently reported hybrid electrolyzers.

Encouraged/Impressed by the fascinating three‐electrode performance of SnSe/SnTe/NF toward HER, OER, and MOR, we further investigated the performance of SnSe/SnTe/NF as a bifunctional electrocatalyst toward conventional electrochemical water splitting (HER + OER) and a hybrid MOR‐mediated water electrolyzer (HER + MOR). **Figure** **4**a shows the two‐electrode performance of SnSe/SnTe/NF as both anodic and cathodic electrodes in 1 m KOH electrolyte with 0.5 m CH_3_OH (HER//MOR) and without CH_3_OH (HER//OER). The comparison performance with bare NF electrodes is also shown in Figure 4a. As depicted in Figure 4a, impressively, SnSe/SnTe/NF as a bifunctional electrode requires 1.54 V, and bare NF requires 1.75 V to achieve 10 mA cm^−2^ in a conventional water electrolyzer (HER//OER). Impressively, when methanol is introduced into the system (HER//MOR), the HWE (SnSe/SnTe/NF//SnSe/SnTe/NF) required only a very less voltage of only 1.49 and 1.69 V to reach 10 and 100 mA cm^−2^, which is 230 mV lesser compared to the conventional electrolyzer to reach current density of 100 mA cm^−2^ and recently reported hybrid and conventional water electrolyzer, as shown in Table S3, Supporting Information. Apart from the energy‐saving performance of HWEs, analyzing the long‐term stability of the catalysts for real‐world applications is essential. Notably, the stability analysis of catalysts is analyzed using chronopotentiometry analysis (Figure 4b) by applying a current density of 100 mA cm^−2^ for 25 h, which explicits the stability of the catalysts with considerable change in the potential; this change can be attributed to the oxidation of methanol. In addition, the inset in Figure 4b reveals no bubbles on the anode's electrode surface, demonstrating that the OER process has been thermodynamically substituted by the MOR. To provide additional confirmation of the catalysts’ stability, we conducted LSV after subjecting the same electrodes to 25 h of continuous operation in a fresh electrolyte with 0.5 m CH_3_OH. The results indicate a negligible change of only 0.02 V to achieve a current density of 100 mA cm^−2^ in the LSV curve (Figure S15, Supporting Information). This underscores the electrode's outstanding performance and further supports our earlier assumption that the observed variations in the chronopotentiometric curve are attributed to the complete oxidation of CH_3_OH. After the stability test, the electrolytic products were investigated using ^1^H NMR measurements, as shown in Figure 4c. Among the various possible C1 products such as formaldehyde, formate etc., we observed only one peak at 8.27 ppm corresponding to the HCOO^−^ whereas the formaldehyde peaks which generally are at 9.66 ppm are absent which verify the selective catalytic conversion of methanol to formate at the anode.^[^ ^13^, ^67^ ^]^ In addition to further analyze the real‐time performance and to investigate the amount of hydrogen production, we constructed an H‐type membrane water electrolyzer using bifunctional SnSe/SnTe/NF as electrodes and an anion exchange membrane (AEM) serving as the separator. Figure 4d shows the schematic representation of H‐type water electrolyzer. The quantity of generated hydrogen was measured using a water drainage method, and the digital photographs of the H‐cell used for the electrolyzer and the drainage setup employed for H_2_ collection are illustrated in Figure 4e inset and in Figure S16, Supporting Information. As a proof of concept, we introduced 0.5 m methanol into the anodic chamber and applied 100 mA cm^−2^ current density to the device. Bubble formation was absent in the anodic electrode, confirming the occurrence of anodic methanol oxidation (Video S1, Supporting Information). Figure 4e presents the H_2_ collected at different time intervals using the drainage setup, and the cathode exhibits a hydrogen production with an amount close to the theoretical value with a Faradaic efficiency of 95%. Overall, the hybrid water electrolysis investigations demonstrate that SnSe/SnTe/NF is a promising candidate for conventional/hybrid water electrolysis systems. Furthermore, the SnSe/SnTe/NF electrodes were tested for practical applicability using 1.0 m KOH with and without 0.5 m CH_3_OH diluted in tap water and natural seawater. Figure 4f depicts that compared to seawater, the performance in tap water is better due to the high purity of tap water, and the seawater contains a high concentration of certain minerals and inorganic salts which hinders the performance of H_2_ production. Finally, as a proof of concept, we demonstrated a real‐time application by integrating wind‐generated electricity to power a HWE with SnSe/SnTe/NF as bifunctional electrodes and 1 m KOH with 0.5 m CH_3_OH as electrolyte. The windmill‐powered SnSe/SnTe/NF water electrolyzer splits water into H_2_ gas bubbles, as seen in the digital snapshot in Figure 4g and video S2, Supporting Information, and one electrode without bubbles indicates the MOR. Figure 4h compares SnSe/SnTe/NF as a bifunctional HWE with other recently reported literatures, which explicits that our synthesized catalysts operate at a lower potential. These studies proposed using nonprecious SnSe/SnTe/NF electrocatalysts for efficient water splitting utilizing wind energy, one of the most cost‐effective approaches for industrial production of H_2_ in real‐world applications.

#### 2.2.5 Commercial Zero‐Gap Anion Exchange Membrane Water Electrolyzer

**Figure 5:**
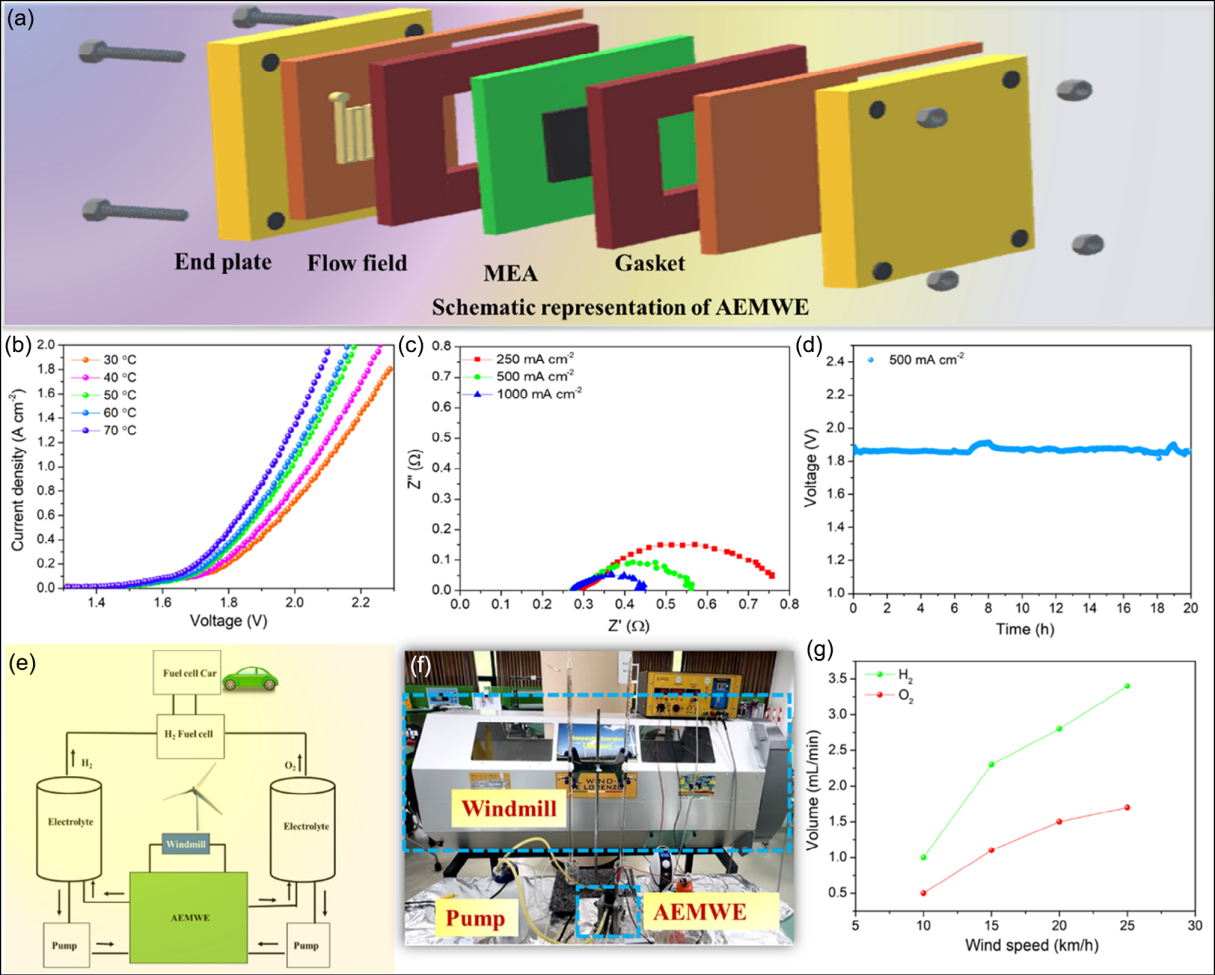
a) Schematic representation of AEMWE with non‐noble SnSe/SnTe/NF electrodes as anode and cathode. b) Polarization curve of AEMWE by applying different temperatures. c) Nyquist curve of AEMWE by applying different current densities. d) Long‐term durability analysis of AEMWE by applying a large current density of 500 mA cm^−2^. e) Flowchart representation of AEMWE powered by windmill. f) Photographic image of windmill‐powered AEMWE. g) Graph representing the wind speed and the hydrogen and oxygen produced.

Further, to guarantee real‐time applications, we assembled a single‐cell anion AEMWE with SnSe/SnTe/NF as anode and cathode catalyst with FUMASEP as a membrane. The various components of the AEMWE cell are shown in the scheme (**Figure** **5**a). The polarization curve of AEMWE at various temperatures using 1 m KOH solutions is demonstrated in Figure 5b. From the polarization curve, it interferes that the SnSe/SnTe/NF AEMWE achieved an industrially required current density of 1 A cm^−2^ at 1.93 V at 70 °C, which is superior to other recently reported literature, as shown in Table S4, Supporting Information. The SnSe/SnTe/NF AEMWE showed better performance at 70 °C compared to 60 °C (1.97 V @ 1 A cm^−2^), 50 °C (1.99 V @ 1 A cm^−2^), 40 °C (2.04 V @ 1 A cm^−2^), 30 °C (2.08 V @ 1 A cm^−2^) due to the increased reaction kinetic of the electrodes and conductivity of the membrane as the temperature increased. It is noteworthy that the zero‐gap AEMWE demonstrated superior performance compared to the membrane‐less single‐chamber electrolyzer. In the AEMWE setup, the most significant reactions occur at the catalyst's surface in a zero‐gap configuration with the anion exchange membrane electrolyte, establishing a three‐phase boundary (electrolyte, solid electrode, and gaseous product). This configuration offers a substantial active area for both mass transfer and charge transfer processes, contributing to the enhanced efficiency observed in the AEMWE system.

Additionally, EIS analysis was conducted by applying various current densities to assess the kinetics of the AEMWE. The results indicate that as the current density increases, both the Ohmic resistance and the charge transfer resistance decrease. This phenomenon is attributed to the dominant activation loss at lower current densities compared to higher ones, as depicted in Figure 5c. Also it is reported that at higher current densities the critical diameter of bubble formation is less and thus the departure of bubble from the surface of the electrode will be faster, resulting in the above trend of Ohmic and charge transfer resistance.^[^ ^68^ ^]^ Moreover, SnSe/SnTe/NF AEMWE also exhibits excellent stability for continuous 18 h at a high current density of 500 mA cm^−2^, which is an indispensable factor for practical demonstrations (Figure 5d). The flowchart to achieve the mission of carbon neutrality using AEMWE is shown in Figure 5e. For real‐time demonstration, we powered the AEMWE with windmill and measured the hydrogen and oxygen production with respect to the wind speed (Figure 5f–g). As the wind speed increases, hydrogen and oxygen production rate increases since more power is supplied to the AEMWE. The overall results demonstrate the excellent performance of a non‐noble SnSe/SnTe/NF as bifunctional AEMWE powered by renewable wind energy as a practical demonstration.

## 3 Conclusion

In summary, for the first time, we have successfully synthesized a non‐noble and cost‐effective electrocatalyst via. in situ growth of tin chalcogenides and tin chalcogenide heterostructure on NF through a facile one‐step hydrothermal technique. Further, we confirm the successful formation of tin chalcogenides and their heterostructures through physiochemical characterization techniques such as XRD, XPS, FESEM, and HRTEM. The three‐electrode measurements reveal the superior performance of heterostructures compared to the single‐phase chalcogenides. Among the three heterostructures, SnSe/SnTe/NF exhibits superior performance, primarily attributed to its rod‐like morphology. This morphology offers a larger aspect ratio, resulting in an increased active surface area. Consequently, there is a more enhanced exposure of active sites, facilitating a more effective electrode–electrolyte interface. This observation is further supported by EIS results. We also demonstrated a commercial zero‐gap anion AEMWE and integrated it with a windmill for real‐world application with the SnSe/SnTe/NF as a bifunctional electrocatalyst. Overall, this work paves the development for unexplored tin chalcogenide heterostructure as a potent candidate to achieve the global mission of carbon neutrality.

## 4 Experimental Section

4.1

4.1.1

##### Chemicals and Reagents

All chemicals utilized in these experiments were of analytical grade and used without further purification. Elemental sulfur and selenium powder, tin chloride, ethanolamine, and potassium hydroxide were purchased from Daejung Chemicals Ltd., South Korea. Elemental tellurium powder was procured from Sigma Aldrich, South Korea. The NF substrate with a thickness of 0.17 cm was procured from Heze Jiaotong Xinda Import and Export Co., Ltd., China.

##### Synthesis of SnSe/SnTe/NF

The synthesis of SnSe/SnTe/NF was carried out using a single‐step hydrothermal process. In brief, 1 mmol Se and 1 mmol Te were added into 30 mL ethanolamine and stirred vigorously for 30 min to form a uniform solution. One mmol of SnCl_2_ was added to the above solution and stirred for another 30 min. Further precleaned NF (2.5 × 2.5 cm^2^) was immersed in the above homogeneous solution and kept for 30 min. Finally, in a 50 mL Teflon‐lined autoclave, the aforementioned solution with pretreatment NF was added and heated to 200 °C for 24 h. Afterward, the autoclave was allowed to cool naturally to room temperature. The retrieved NF was washed multiple times with deionized (DI) water and ethanol and then subjected to drying in a vacuum oven at 60 °C overnight. Similarly, the SnSe/SnS/NF and SnS/SnTe/NF were prepared through the same procedure by adding the appropriate precursors (Se and S) and (S and Te), respectively. Prior to synthesis, the native surface oxide layers and dust were removed from the Ni foam (2.5 × 2.5 cm^2^) by cleaning it with diluted acetone, HCl, and DI water using an ultrasonication method.

##### Physiochemical Characterization

The X‐ray diffractometer (XRD) was employed to investigate the phase purity and crystallinity of the catalysts. This analysis was conducted on an Empyrean XRD (Malvern Panalytical, UK) provided with Cu K radiation (*λ* = 1.54184). To examine surface morphology and elemental mapping of the samples, a FESEM (TESCAN, MIRA3) combined with an EDX analyzer was utilized. Moreover, the heterostructure was assessed using field‐emission transmission electron microscopy (JEOL, JEM‐2100F) at the Korea Basic Science Institute (KBSI) (Daegu, SK). For insights into the surface composition and electronic state of elements within the sample, X‐ray photoelectron spectroscopy (XPS) analysis was implemented using an ESCA‐2000 (VG Microtech Ltd) at the Korea Basic Science Institute (KBSI), Busan, SK.

##### Electrochemical Characterization

The three‐electrode performances for HER, OER, and MOR were investigated using an Autolab PGSTAT302N electrochemical workstation. All electrochemical measurements were performed without special gas pretreatments in the alkaline medium. The Pt sheet, Ag/AgCl and resultant catalyst on NF served as the counter, reference, and working electrode in the three‐electrode measurements, and all the working potential was calibrated to a reversible hydrogen electrode (RHE) using the Nernst equation given below.
(1)

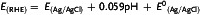




To ensure stability and to avoid polarization interference to a reasonable extent, all the electrocatalysts were activated in 1 m KOH electrolyte by cycling for several cycles before all the measurements. All the current densities were normalized to the geometric area of NF. Linear sweep voltammetry (LSV) curves for HER and OER were carried out in 1 m KOH electrolyte at a scan rate of 5 mV s^−1^. LSV for MOR was carried out in 1 m KOH + 0.5 m CH_3_OH electrolyte. The electrochemical impedance spectroscopy (EIS) analysis was performed by applying a bias potential of −0.227, 1.57, and 1.32 V versus RHE for HER, OER, and MOR, respectively, recorded from 100 kHz to 0.1 Hz with an amplitude of 10 mV. The two electrode measurements were carried out using the same electrode in the cathode and anode in 1 m KOH containing 0.5 m CH_3_OH. Stability analysis of the device was performed by applying the chronopotentiometric technique at 10 mA cm^−2^ for 25 h in 1 m KOH containing 0.5 m CH_3_OH. All the polarization curves were without iR correction. The AEMWE was prepared by sandwiching the FUMASEP membrane between the heterostructured catalysts grown on NF (1 × 1 cm^2^). Subsequently, electrochemical experiments were conducted by circulating 1 m KOH into the cathode and anode using a circulator pump and feeding 20 mL min^−1^. The steady‐state polarization measurements and stability were carried out using Arbin Instruments (USA).

## Conflict of Interest

The authors declare no conflict of interest.

## Supporting information

Supplementary Material

## Data Availability

The data that support the findings of this study are available from the corresponding author upon reasonable request.
