# Supplementary material for: Harnessing Wind Energy for Ultraefficient Green Hydrogen Production with Tin Selenide/Tin Telluride Heterostructures
Source: Small Sci. 2024 Jan 14;4(3):2300222. doi: 10.1002/smsc.202300222 (PMC11935093; doi:10.1002/smsc.202300222)
Supplement: Supplementary file 1 — Supplementary Material [file SMSC-4-2300222-s001.zip › smsc.202300222-sup-0001-suppdata-S1.pdf]

Electronic Supporting Information for

Harnessing wind energy for ultra-efficient green hydrogen production with tin selenide/tin telluride heterostructures

Aparna Sajeev[1], Muthukumar Perumalsamy[1], Vijaykumar Elumalai[1], Arunprasath Sathyaseelan[1], Saj Anandhan Ayyappan[1], Monunith Anithkumar [1], and Sang-Jae Kim[1,2,3,*]

[1]Nanomaterials & System Lab, Major of Mechatronics Engineering,

Faculty of Applied Energy System, Jeju National University, Jeju 63243, South Korea.

[2]Nanomaterials & System Lab, Major of Mechanical System Engineering, College of Engineering, Jeju National University, Jeju 63243, South Korea.

[3] Research Institute of Energy New Industry (RINEI), Jeju National University, Jeju 63243, South Korea.

*Corresponding author Email: kimsangj@jejunu.ac.kr

1. Calculation for hydrogen production

The faradaic efficiency of the hybrid water electrolyser device was calculated by water drainage method using lab made H-type membrane water electrolyser device.The amount of hydrogen gas (H_2) evolved during the electrolysis process was collected through measuring cylinder by applying a constant current density with various time interval. The amount of hydrogen released as theoretical were calculated using following faraday's law.

$$V_{Theo} = IRTt/PzF \qquad \text{----(1)}$$

V_{Theo} = Theoretical volume of evolved gas

I= working current density (mA cm^{-2})

T is working temperature (K) and 't' is time interval (s)

R is the gas constant and 'P' is the working pressure

F is the Faraday's constant (F=96485 C)

z is the number of electrons for generating 1 mol H_2 (z = 2)

Faradaic efficiency (ηF) was determined by ratio of measured gas volume (V_{meas}) and theoretically calculated volumes (V_{Theo}) as given in equation

$$\eta F = V_{meas}/V_{Theo} \qquad \text{------(2)}$$

Mechanism of hydrogen evolution reaction in the alkaline medium

Mechanism of hydrogen evolution reaction occurring at the surface of prepared catalysts are given below.

$H_2O + e^- + * \rightarrow H^* + HO^-$ (3)

Volmer (120 mV dec^{-1})

$H_2O + e^- + H^* \rightarrow H_2 + HO^-$ (4)

Heyrovsky (40 mV dec^{-1})

$H^* + H^* \rightarrow H_2$ (5)

Tafel (30 mV dec^{-1})

Mechanism of oxygen evolution reaction in the alkaline medium

Mechanism of oxygen evolution reaction occurring at the surface prepared catalysts are given below.

$* + OH^- \rightarrow *OH^- + e^-$ (6)

(Tafel slope: 120 mV dec^{-1})

$*OH + OH \rightarrow *O^{-} + H_2O$ (7)

(Tafel slope: 60 mV dec^{-1})

$*O^{-} \rightarrow *O + e-$ (6) (8)

(Tafel slope: 40 mV dec^{-1})

$2*O \rightarrow O_2\uparrow$ (9)

(Tafel slope: 15 mV dec^{-1})

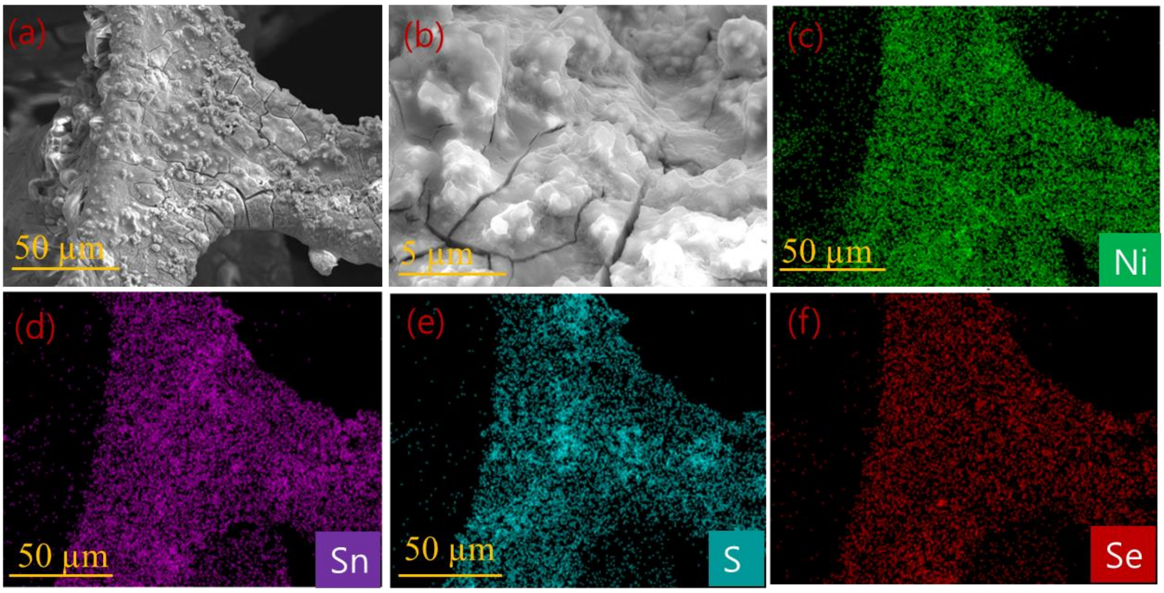

Figure S1. FE-SEM and EDX analysis of SnSe/SnS

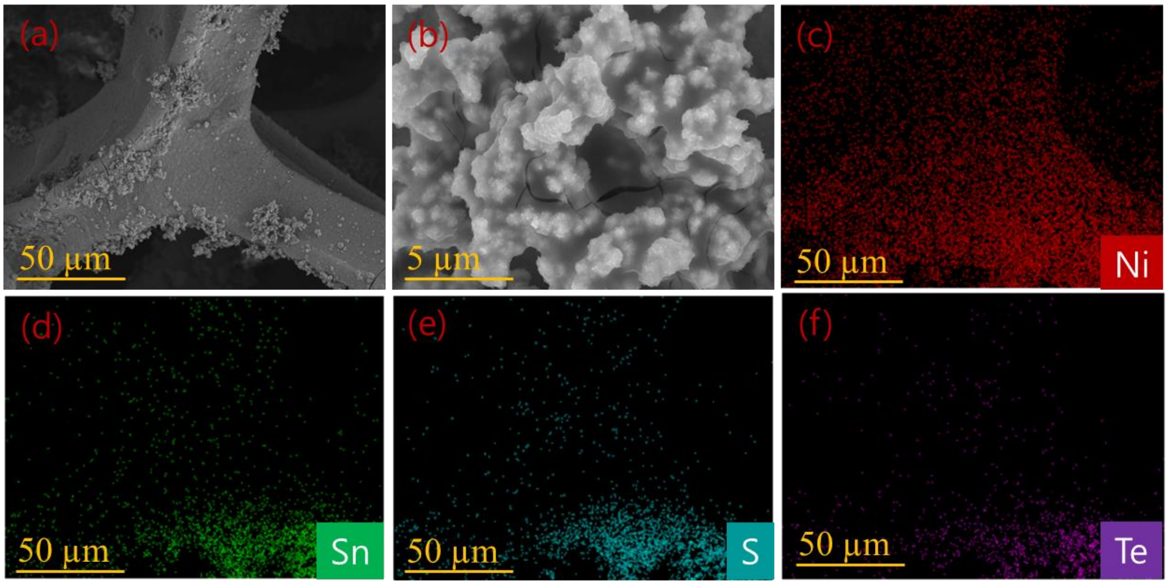

Figure S2. FE-SEM and EDX analysis of SnS/SnTe

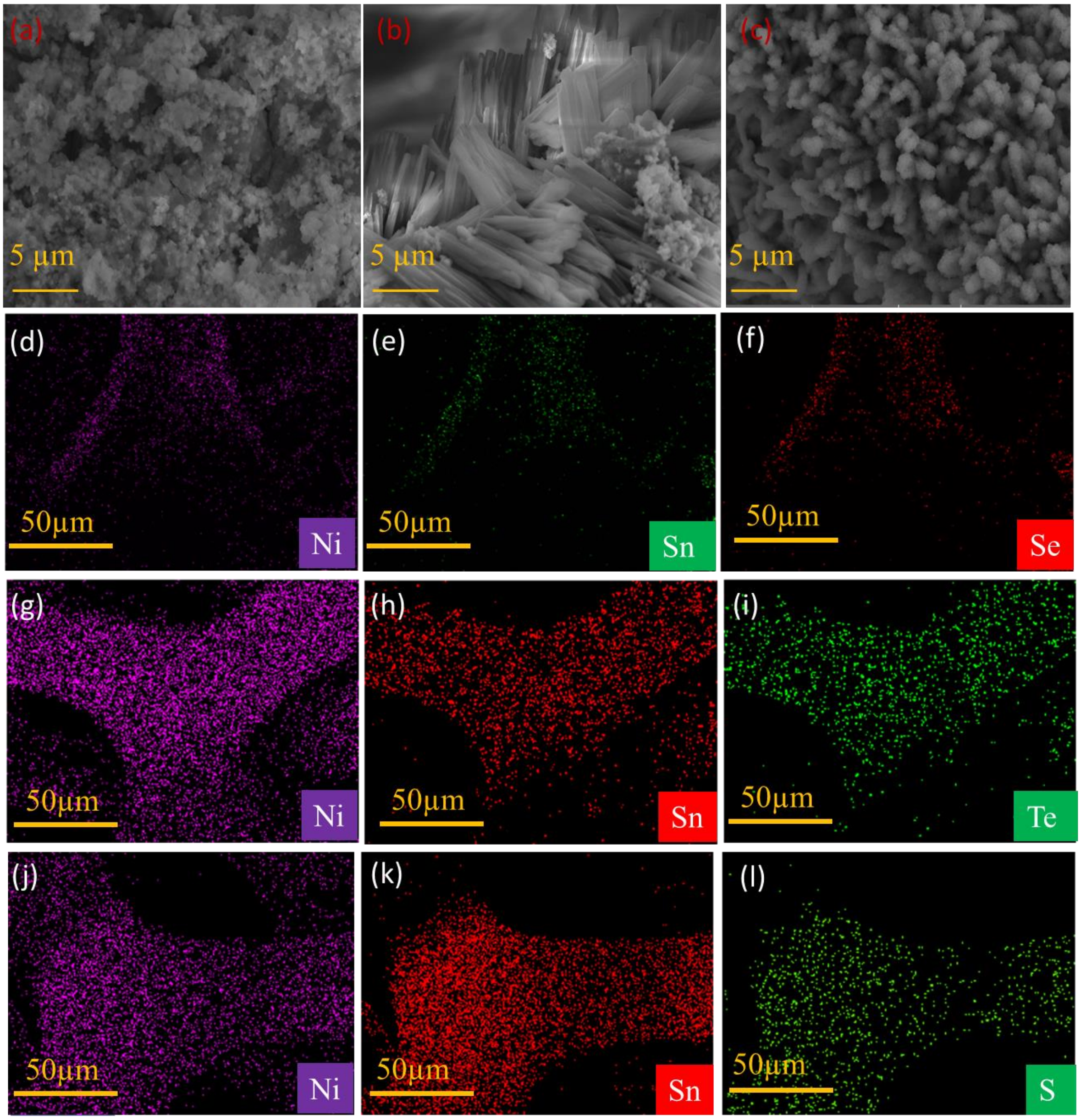

Figure S3. FE-SEM analysis of (a) SnSe (b) SnTe (c) SnS. EDX analysis of (d-f) SnSe (g-i) SnTe and (j-l) SnS

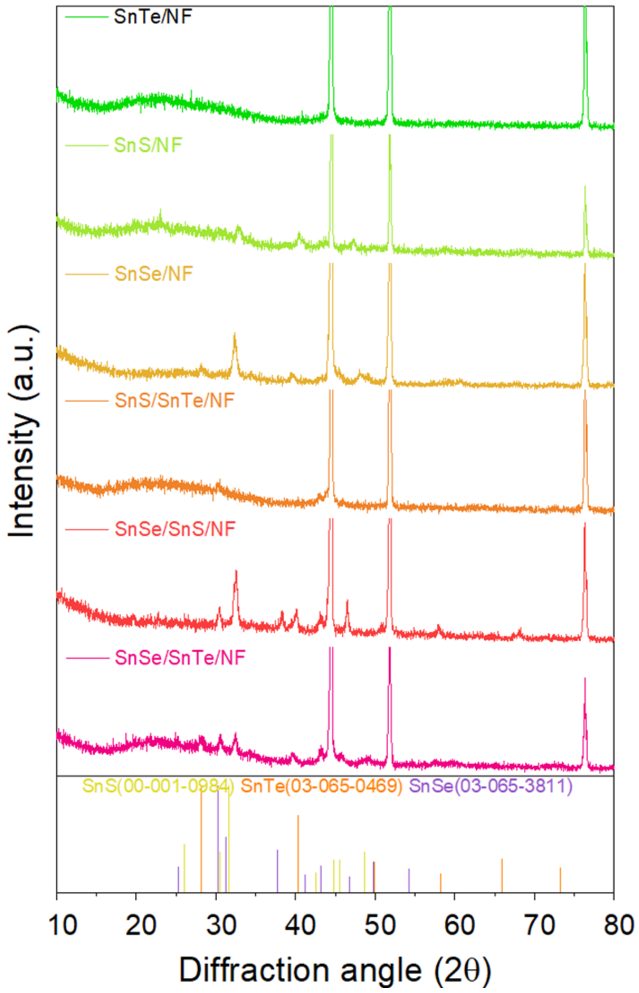

Figure S4. XRD spectra of all samples on NF

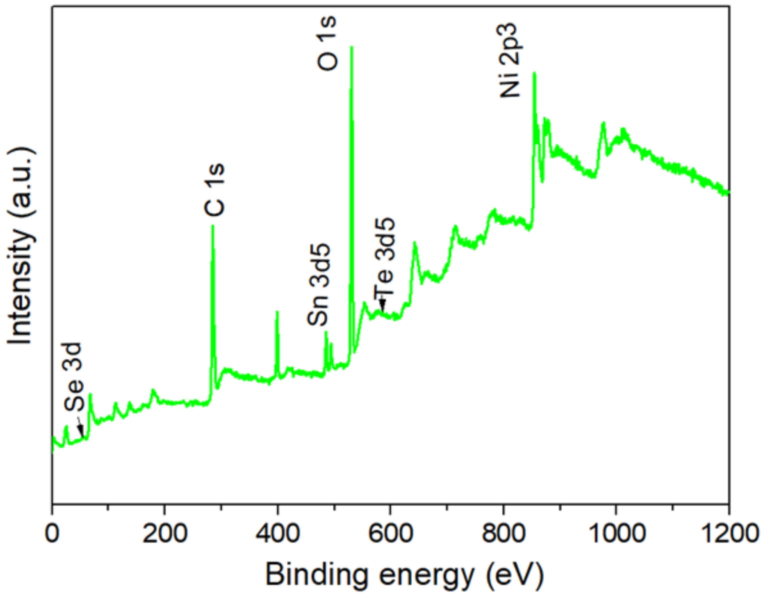

Figure S5. XPS survey spectrum of SnSe/SnTe/NF

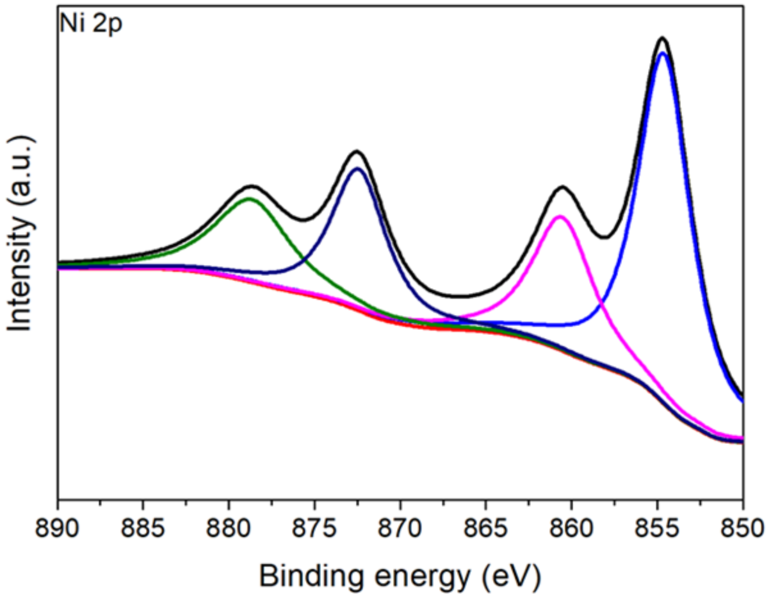

Figure S6. Deconvoluted XPS spectra of Ni 2p

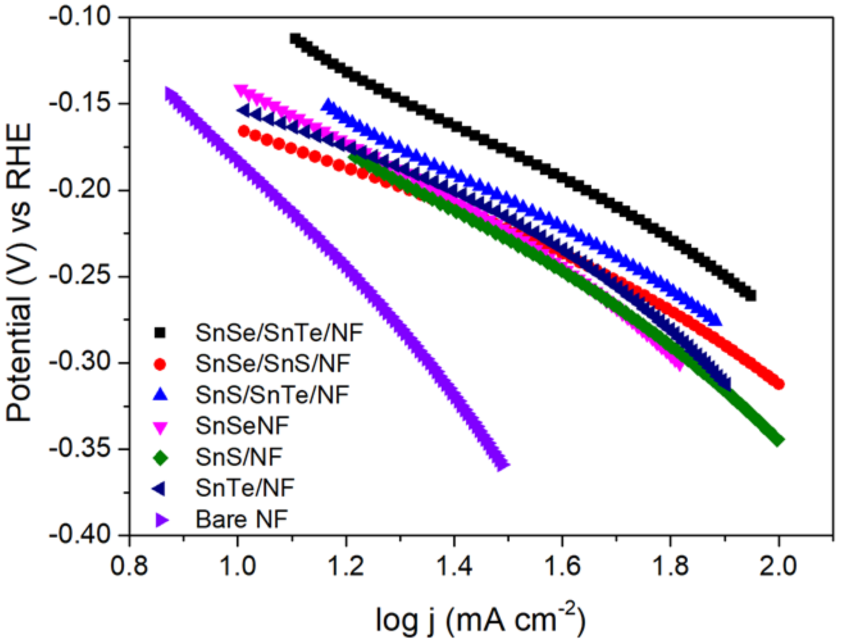

Figure S7. Tafel analysis of all catalysts towards HER

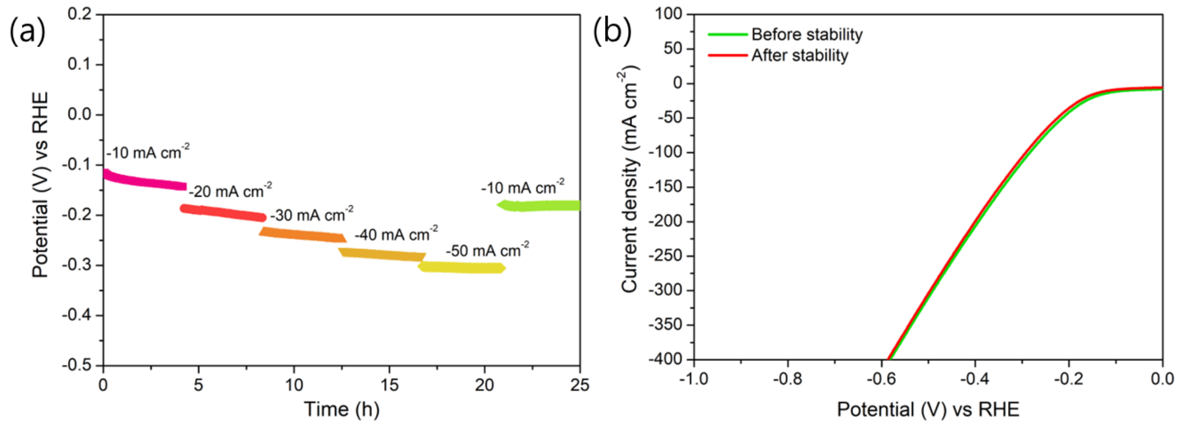

Figure S8. (a) multi-potential analysis of SnSe/SnTe/NF electrodes for 25 h by applying multiple currents (b) LSV polarization curve before and after stability for HER

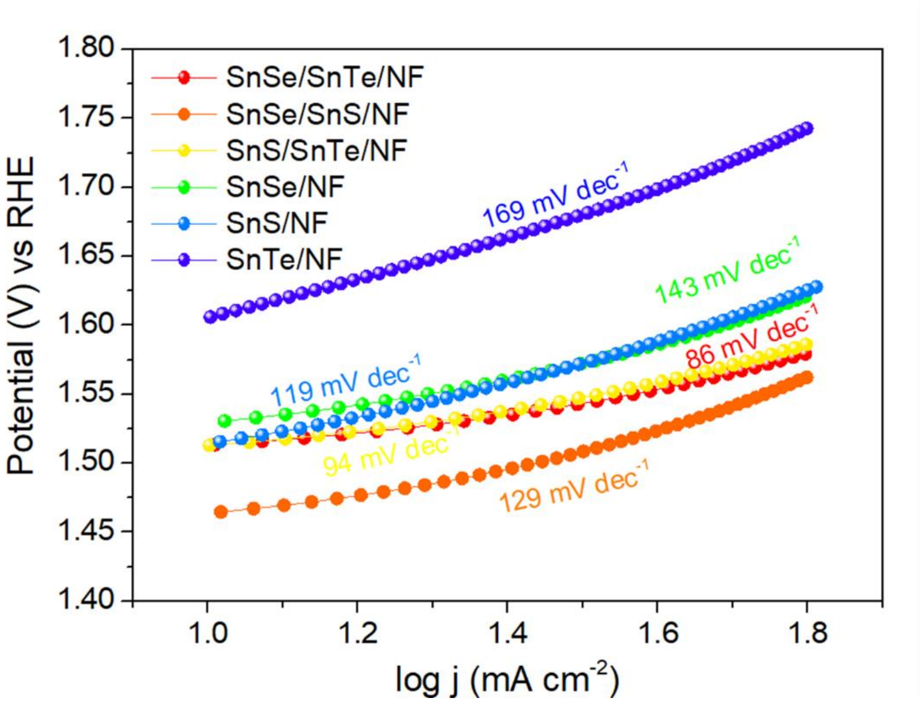

Figure S9. Tafel analysis of all catalysts towards OER

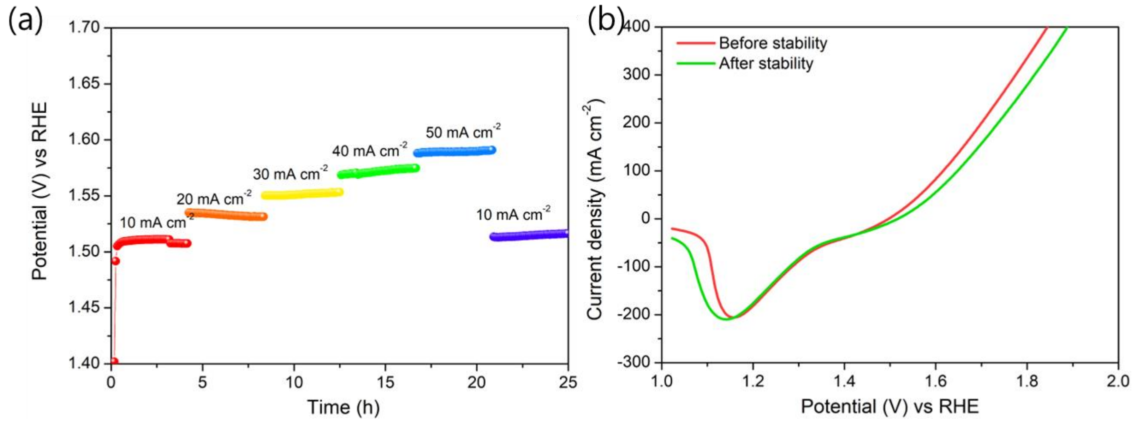

Figure S10. (a) multi-potential analysis of SnSe/SnTe/NF electrodes for 25 h by applying multiple currents (b) LSV polarization curve before and after stability for OER

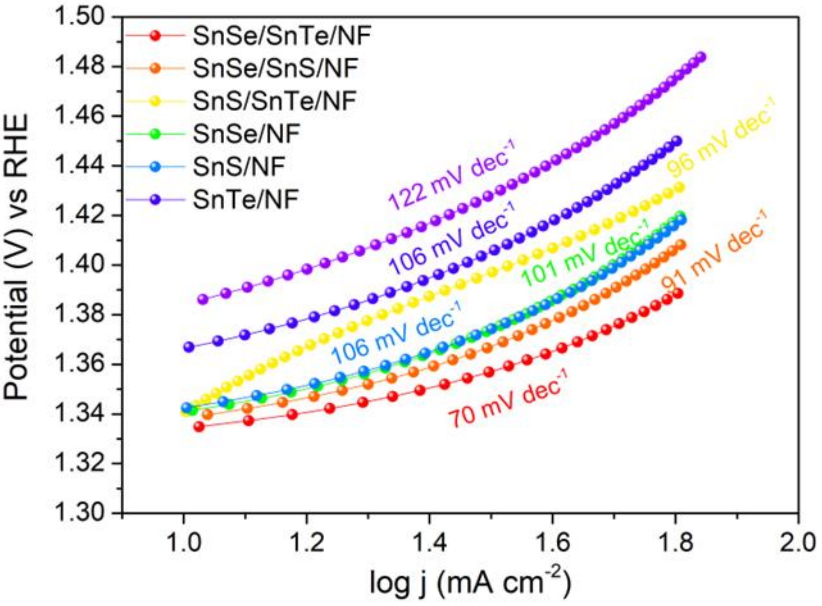

Figure S11. Tafel analysis of all catalysts towards MOR

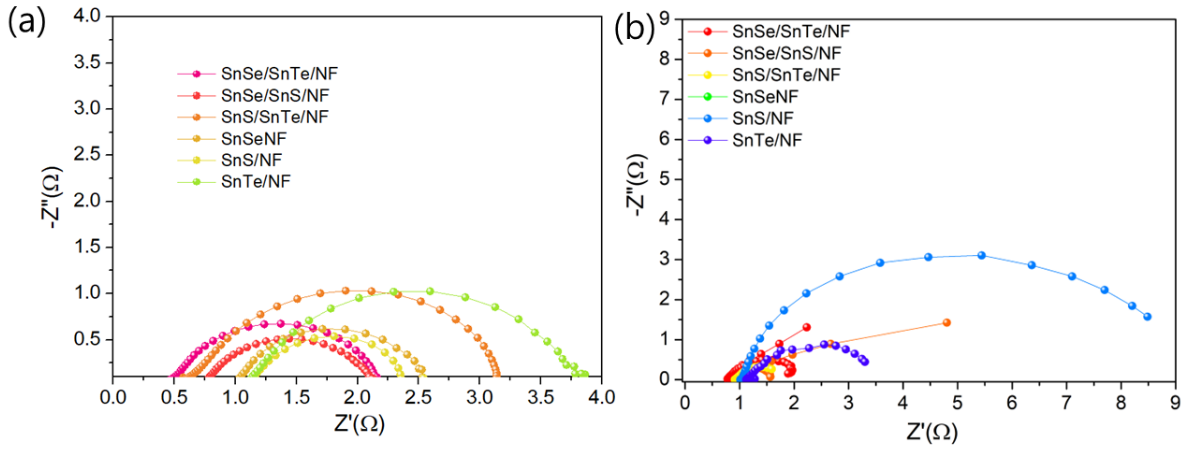

Figure S12. EIS analysis of various catalysts by applying a bias potential of (a)-0.22 V and (b) 1.57 V vs. RHE

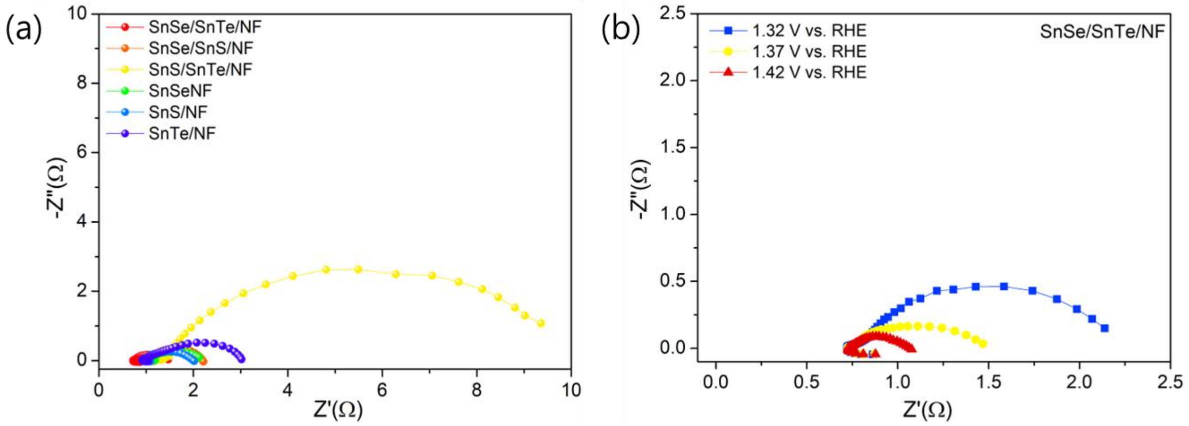

Figure S13. Nyquist plot of all catalysts by applying a bias potential of 1.32 V vs RHE. (f) Nyquist plot of SnSe/SnTe/NF by applying different bias potential of 1.32 V, 1.37 V and 1.42 V vs RHE for MOR

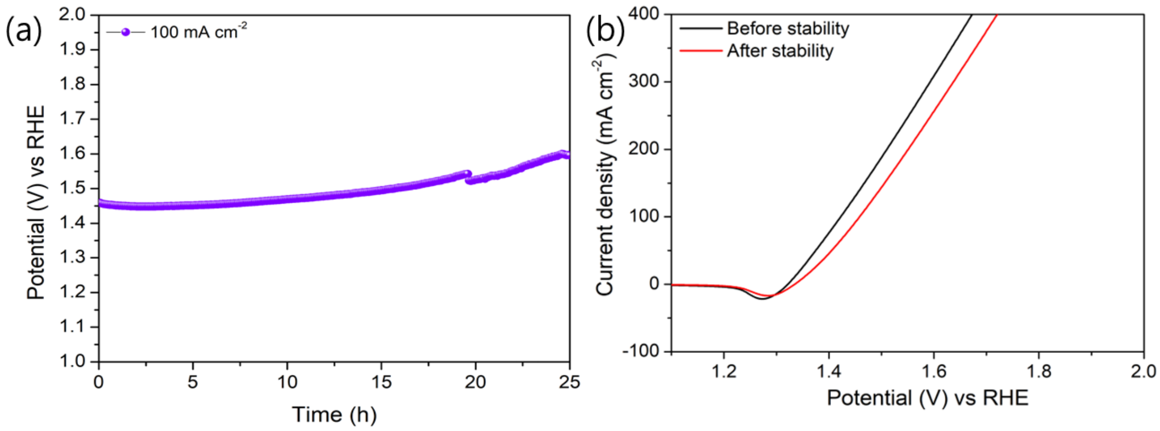

Figure S14.Stability measurement of SnSe/SnTe/NF up to 25 h for MOR by applying a constant current of 100 mA cm^{-2}(b) LSV polarization curve before and after stability for MOR

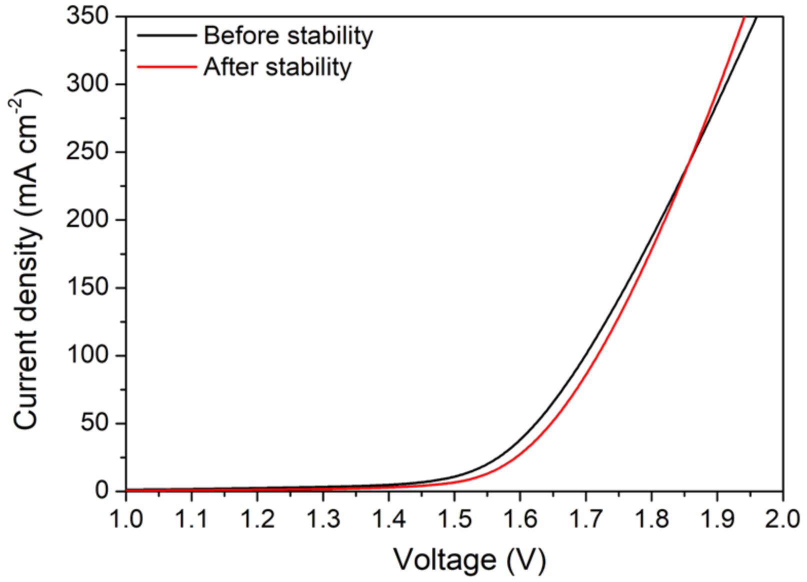

Figure S15. LSV analysis of SnSe/SnTe// SnSe/SnTe in 1 M KOH + 0.5 M CH_3OH before and after continuous operation of 25 h

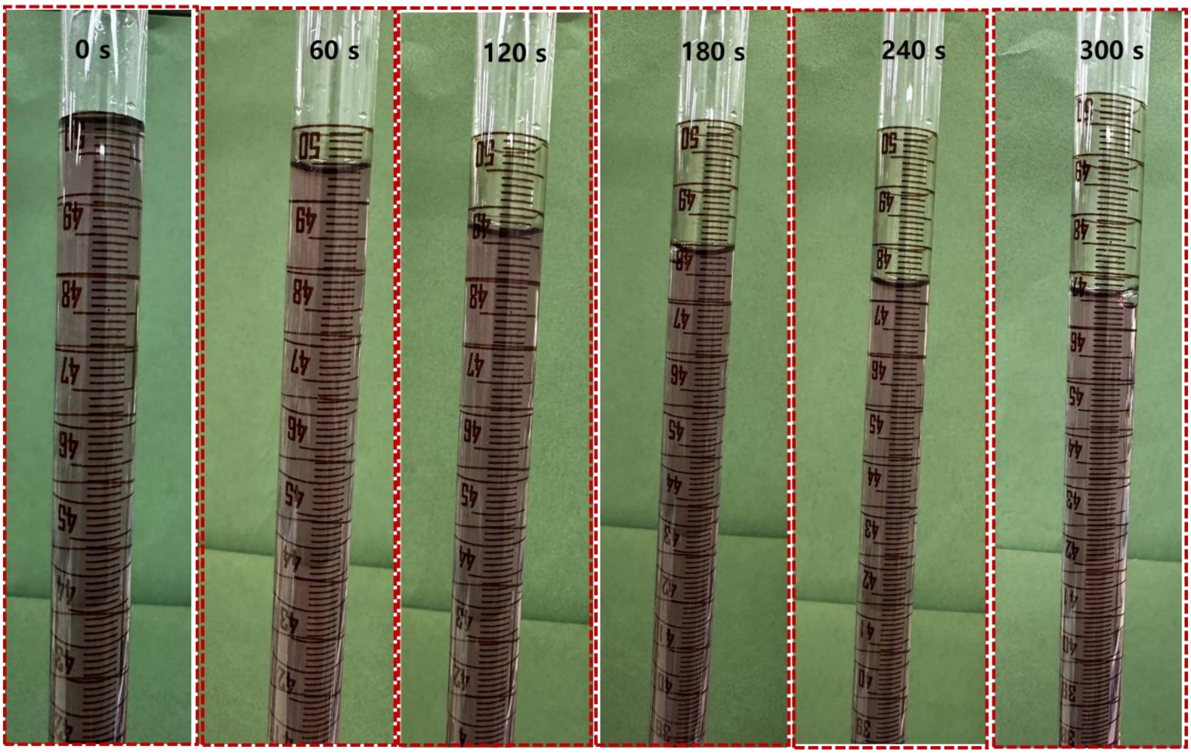

Figure S16. Photograph of water drainage method produced hydrogen at regular intervals

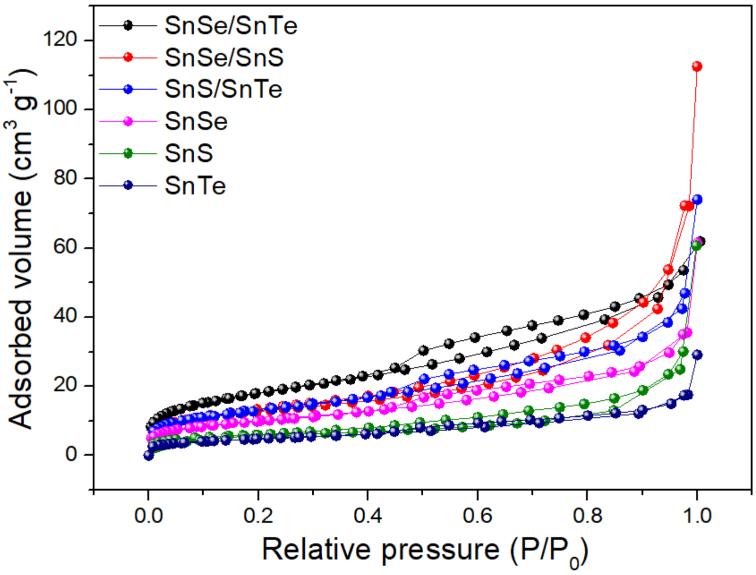

Figure S17. BET N_2 adsorption-desorption isotherm of all prepared catalysts.

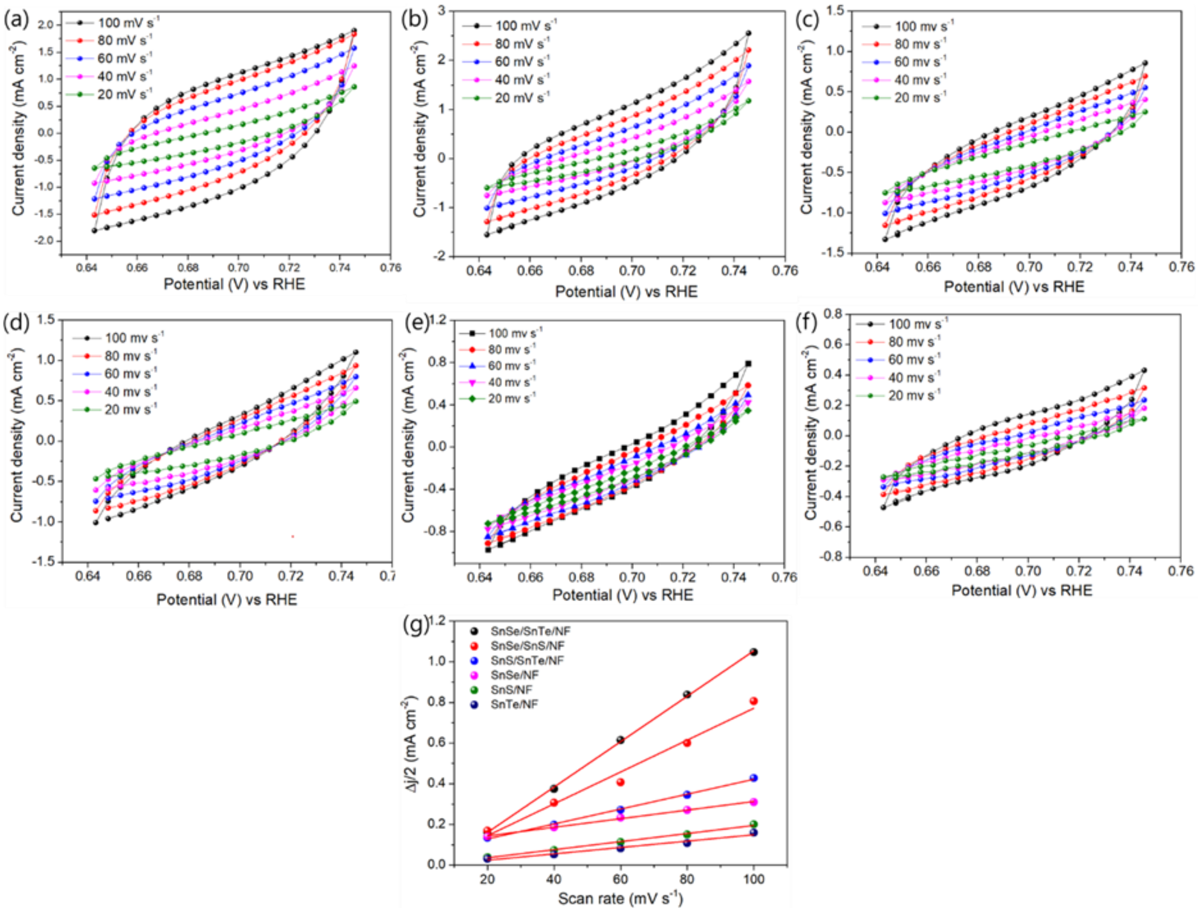

Figure S18. Electrochemical double-layer capacitance measurements of (a) SnSe/SnTe/NF, (b) SnSe/SnS/NF, (c) SnS/SnTe/NF, (d) SnSe/NF, € SnS/NF, (f) SnTe/NF at scan rates of 20, 40, 60, 80 and 100mV s-1 (g) The plot of scan rate vs current density of all the prepared samples at 0.7065 V vs. RHE.

Table S1: Comparison analysis of SnSe/SnTe/NF heterostructured catalysts towards HER with recent reported literatures

Sl no	Catalyst	Electrolyte	Current density(mA cm^{-2})	Overpotential (mV)	Reference
1	SnSe/SnTe/NF	1 M KOH	10	77	This work
2	$(Fe,Ni)S_2$@MoS_2/NiS_2	1 M KOH	50	176	[1]
3	SCNO@N-CNF	1 M KOH	10	169	[2]
4	WSe_2@$SnSe_2$	1 M KOH	10	180	[3]
5	$Co_{1.11}Te_2/Te$	1 M KOH	10	135	[4]
6	$CoSe/Co(OH)_2$-CM	1 M KOH	10	207	[5]
7	SnO_2@MoS_2	1 M KOH	10	127	[6]
8	SnO_2@g-C_3N_4@SnS_2	1 M KOH	10	386	[7]
9	2D SnS-Ni_3S_2	1 M KOH	10	145	[8]
10	$CuInS_2/SnS_2$/NF	1 M KOH	10	156.9	[9]
11	$NiCo_2Se_4/NiCoS_4$	1 M KOH	10	180	[10]
12	CuNi@NiSe	1 M KOH	10	41	[11]

Table 2: Comparison analysis of SnSe/SnTe/NF heterostructured catalysts towards OER with recent reported literatures

Sl no	Catalyst	Electrolyte	Current density(mA cm^{-2})	Overpotential (mV)	Reference
1	SnSe/SnTe/NF	1 M KOH	10	281	This work
2	$(Fe,Ni)S_2$@MoS_2/NiS_2	1 M KOH	50	342	[1]
3	SCNO@N-CNF	1 M KOH	10	247	[2]
4	WSe_2@$SnSe_2$	1 M KOH	10	250	[3]
5	$Co_{1.11}Te_2/Te$	1 M KOH	10	261	[4]
6	$CoSe/Co(OH)_2$-CM	1 M KOH	10	299	[5]
7	SnO_2@MoS_2	1 M KOH	50	270	[6]
8	SnO_2@g-C_3N_4@SnS_2	1 M KOH	10	475	[7]
9	2D SnS-Ni_3S_2	1 M KOH	20	298	[8]
10	Cu-(a-$NiSe_x$/c-$NiSe_2$)/TiO_2	1 M KOH	10	339	[9]
11	$NiCo2Se_4/NiCoS_4$	1 M KOH	10	248	[10]
12	CuNi@NiSe	1 M KOH	10	293	[11]

Table 3: Comparison analysis of SnSe/SnTe/NF heterostructured catalysts towards MOR with recent reported literatures

Sl no	Catalyst	Electrolyte	Current density(mA cm^{-2})	Overpotential (V)	Reference
1	SnSe/SnTe/NF	1 M KOH +0.5M CH_3OH	10	1.33	This work
2	P-$CoNi_2S_4$	1 M KOH +0.5M Ch_4N_2O	10	1.306	[12]
	Ni-MOFs-120/NF	1 M KOH +0.5M CH_3OH	10	1.37	[13]
4	$Co(OH)_2$@HOS/CP	1 M KOH + 1M CH_3OH	10	1.39	[14]
5	Ni MOF/NF	1 M KOH + 4M CH_3OH	10	1.39	[15]
6	NiCoO-400	1 M KOH + 1M CH_3OH	10	1.4	[16]
7	NiO/NF	1 M KOH + 1M CH_3OH	10	1.38	[17]
8	NiS/NF	1 M KOH + 1M CH_3OH	10	1.36	[18]
9	$Ni_{1.7}Sn$ NPs/CB	0.5 M KOH + 0.5M CH_3OH	10	1.48	[19]
10	$Ni_{2.5}Co_{0.5}Sn_2$ NPs	1 M KOH + 1M CH_3OH	10	1.45	[20]
11	NiFe NPs	1 M KOH + 1M CH_3OH	10	1.53	[21]
12	$NiSe_2$ on NiAl LDH	1 M KOH + 0.5M CH_3OH	10	1.37	[22]

Table 4: Comparison analysis of SnSe/SnTe/NF heterostructured catalysts towards hybrid water electrolyzers

Sl no	Catalyst	Electrolyte	Current density(mA cm^{-2})	Voltage (V)	Reference
1	SnSe/SnTe/NF	1 M KOH +0.5M CH_3OH	10	1.49	This work
2	P-$CoNi_2S_4$	1 M KOH, 0.5 M Ch_4N_2O	10	1.402	[12]
3	NP-NiFe (NP-Ni0.7Fe0.3)	1 M KOH, 0.33 M Ch_4N_2O	10	1.55	[23]
4	Co_9S_8@MoS_2	1 M KOH +0.5M CH_3OH	10	1.49	[24]
5	α-$Co(OH)_2$/CP	1 M KOH + 3M CH_3OH	10	1.758	[25]
6	Ni_2P/NF	1.M KOH+10 mM HMF	10	1.65	[26]
7	Co-S-P/CC	1 M KOH +1 M C_2H_6OH	10	1.63	[27]
8	$Co(OH)_2$@HOS/CP	1 M KOH + 3M CH_3OH	10	1.497	
9	Ni $(OH)_2$/NF	1 M KOH + 0.5M CH_3OH	10	1.52	[28]
10	$Ni_{0.33}Co_{0.67}(OH)_2$/NF	1 M KOH + 0.5 CH_3OH	10	1.50	[29]
11	Co-Rh_2	1 M KOH + 1M methanol	10	1.545	[30]
12	Ni-Se/NF	1 M KOH, 0.33 M Ch_4N_2O	10	1.41	[31]

Table 5: Comparison analysis of SnSe/SnTe/NF heterostructured catalysts AEMWE device with recent reported literatures

Sl no	Catalyst	Current density(mA cm^{-2})	Voltage (V)	Temperature (°C)	Reference
1	SnSe/SnTe/NF	500<br>1000	1.81<br>1.93	70	This work
	SnSe/SnTe/NF	500<br>1000	1.84<br>1.97	60	This work
2	NiFeS@Ti_3C_2 MXene/NF	401	1.85	50	[32]
3	Co, Mo-NiFe LDH	400	1.91		[33]
4	$Co_2Mo_3O_8/MoO_2$/NF	200	2.53		[34]
5	$Pt_2/Ni(OH)_2$/NF	10	1.46		[35]
6	Ni_2P/Ni_7S_6//Pt/C	1000	1.99		[36]
7	NiFe//NiMo	500	1.87		[37]
8	IrO_2 // Pt/C	320	1.8	60	[38]
9	Ni//Ni	150	1.9	50	[39]
10	NiCoO-NiCo/C //$Cu_{0.75}Co_{2.2}O_4$	503	1.85	60	[40]

References

[1] L. Tong *et al.*, "(Fe, Ni)S2@MoS2/NiS2 hollow heterostructure nanocubes for high-performance alkaline water electrolysis," *Int J Hydrogen Energy*, vol. 47, no. 21, pp. 11143–11152, Mar. 2022, doi: 10.1016/J.IJHYDENE.2022.01.161.

[2] S. Surendran *et al.*, "Sulphur Assisted Nitrogen-Rich CNF for Improving Electronic Interactions in Co-NiO Heterostructures Toward Accelerated Overall Water Splitting (Adv. Mater. Technol. 2/2023)," *Adv Mater Technol*, vol. 8, no. 2, p. 2370007, Jan. 2023, doi: 10.1002/ADMT.202370007.

[3] M. Velpandian, G. Ummethala, S. K. Malladi, and P. Meduri, "Heterostructures of tin and tungsten selenides for robust overall water splitting," *J Colloid Interface Sci*, vol. 623, pp. 561–573, Oct. 2022, doi: 10.1016/J.JCIS.2022.05.052.

[4] G. Wang, C. Hua, W. Chen, H. Fan, P. Feng, and Y. Zhu, "Intriguing 3D micro-flower structure of Co1.11Te2 deposited on Te nanosheets showing an efficient bifunctional electrocatalytic property for overall water splitting," *Electrochim Acta*, vol. 447, p. 142133, Apr. 2023, doi: 10.1016/J.ELECTACTA.2023.142133.

[5] C. Gong *et al.*, "Interfacial engineering of ZIF-67 derived CoSe/Co(OH)2 catalysts for efficient overall water splitting," *Compos B Eng*, vol. 236, p. 109823, May 2022, doi: 10.1016/J.COMPOSITESB.2022.109823.

[6] W. He *et al.*, "SnO2@MoS2 heterostructures grown on nickel foam as highly efficient bifunctional electrocatalyst for overall water splitting in alkaline media," *J Alloys Compd*, vol. 938, p. 168678, Mar. 2023, doi: 10.1016/J.JALLCOM.2022.168678.

[7] B. Zhang, J. Li, Q. Song, S. Lv, Y. Shi, and H. Liu, "g-C3N4-modulated bifunctional SnO2@g-C3N4@SnS2 hollow nanospheres for efficient electrochemical overall water splitting," *Appl Surf Sci*, vol. 589, p. 153016, Jul. 2022, doi: 10.1016/J.APSUSC.2022.153016.

[8] X. Zang, J. Teng, X. Zhang, and J. Guo, "Heterogeneous SnS-Ni3S2 nanostructure for efficient overall water splitting," *Mater Lett*, vol. 287, p. 129290, Mar. 2021, doi: 10.1016/J.MATLET.2020.129290.

[9] J. Yuan *et al.*, "A Superaerophobic Bimetallic Selenides Heterostructure for Efficient Industrial-Level Oxygen Evolution at Ultra-High Current Densities," *Nanomicro Lett*, vol. 12, no. 1, pp. 1–12, May 2020, doi: 10.1007/S40820-020-00442-0/FIGURES/5.

[10] K. Wang *et al.*, "Selenide/sulfide heterostructured NiCo2Se4/NiCoS4 for oxygen evolution reaction, hydrogen evolution reaction, water splitting and Zn-air batteries," *Electrochim Acta*, vol. 368, p. 137584, Feb. 2021, doi: 10.1016/J.ELECTACTA.2020.137584.

[11] D. Cao *et al.*, "Interfacial Engineering of Copper–Nickel Selenide Nanodendrites for Enhanced Overall Water Splitting in Alkali Condition," *Small*, p. 2301613, 2023, doi: 10.1002/SMLL.202301613.

[12] X. F. Lu, S. L. Zhang, W. L. Sim, S. Gao, and X. W. (David) Lou, "Phosphorized CoNi2S4 Yolk-Shell Spheres for Highly Efficient Hydrogen Production via Water and Urea Electrolysis," *Angewandte Chemie*, vol. 133, no. 42, pp. 23067–23073, Oct. 2021, doi: 10.1002/ANGE.202108563.

[13] J. Li, "Nickel-organic frameworks as highly efficient catalyst for electrochemical conversion of CH3OH into formic acid," *Electrochem commun*, vol. 146, p. 107416, Jan. 2023, doi: 10.1016/J.ELECOM.2022.107416.

[14] K. Xiang *et al.*, "Boosting H2 Generation Coupled with Selective Oxidation of Methanol into Value-Added Chemical over Cobalt Hydroxide@Hydroxysulfide Nanosheets Electrocatalysts," *Adv Funct Mater*, vol. 30, no. 10, p. 1909610, Mar. 2020, doi: 10.1002/ADFM.201909610.

[15] Y. Xu *et al.*, "Methanol electroreforming coupled to green hydrogen production over bifunctional NiIr-based metal-organic framework nanosheet arrays," *Appl Catal B*, vol. 300, p. 120753, Jan. 2022, doi: 10.1016/J.APCATB.2021.120753.

[16] S. Chen *et al.*, "Hollow and porous NiCo2O4 nanospheres for enhanced methanol oxidation reaction and oxygen reduction reaction by oxygen vacancies engineering," *Appl Catal B*, vol. 291, p. 120065, Aug. 2021, doi: 10.1016/J.APCATB.2021.120065.

[17] M. I. Abdullah, A. Hameed, N. Zhang, M. H. Islam, M. Ma, and B. G. Pollet, "Ultrasonically Surface-Activated Nickel Foam as a Highly Efficient Monolith Electrode for the Catalytic Oxidation of Methanol to Formate," *ACS Appl Mater Interfaces*, vol. 13, no. 26, pp. 30603–30613, Jul. 2021, doi: 10.1021/ACSAMI.1C06258/SUPPL_FILE/AM1C06258_SI_001.PDF.

[18] L. Wei *et al.*, "Integrating hydrogen production with selective methanol oxidation to value-added formate over a NiS bifunctional electrocatalyst," *IOP Conf Ser Earth Environ Sci*, vol. 651, no. 4, p. 042062, Feb. 2021, doi: 10.1088/1755-1315/651/4/042062.

[19] J. Li *et al.*, "NiSn bimetallic nanoparticles as stable electrocatalysts for methanol oxidation reaction," *Appl Catal B*, vol. 234, pp. 10–18, Oct. 2018, doi: 10.1016/J.APCATB.2018.04.017.

[20] J. Li *et al.*, "Colloidal Ni–Co–Sn nanoparticles as efficient electrocatalysts for the methanol oxidation reaction," *J Mater Chem A Mater*, vol. 6, no. 45, pp. 22915–22924, Nov. 2018, doi: 10.1039/C8TA08242A.

[21] S. L. Candelaria *et al.*, "Multi-Component Fe-Ni Hydroxide Nanocatalyst for Oxygen Evolution and Methanol Oxidation Reactions under Alkaline Conditions," *ACS Catal*, vol. 7, no. 1, pp.

365–379, Jan. 2017, doi: 10.1021/ACSCATAL.6B02552/SUPPL_FILE/CS6B02552_SI_001.PDF.

[22] X. Wang, J. Wang, A. Xu, Y. Chang, J. Jia, and M. Jia, "Effect of in situ growth of NiSe2 on NiAl layered double hydroxide on its electrocatalytic properties for methanol and urea," *Int J Hydrogen Energy*, vol. 48, no. 58, pp. 22060–22068, Jul. 2023, doi: 10.1016/J.IJHYDENE.2023.03.040.

[23] Z. Cao *et al.*, "Hydrogen Production from Urea Sewage on NiFe-Based Porous Electrocatalysts," *ACS Sustain Chem Eng*, vol. 8, no. 29, pp. 11007–11015, Jul. 2020, doi: 10.1021/ACSSUSCHEMENG.0C04049/SUPPL_FILE/SC0C04049_SI_001.PDF.

[24] L. He *et al.*, "Multicomponent Co9S8@MoS2 nanohybrids as a novel trifunctional electrocatalyst for efficient methanol electrooxidation and overall water splitting," *J Colloid Interface Sci*, vol. 586, pp. 538–550, Mar. 2021, doi: 10.1016/J.JCIS.2020.10.119.

[25] K. Xiang *et al.*, "Boosting H2 Generation Coupled with Selective Oxidation of Methanol into Value-Added Chemical over Cobalt Hydroxide@Hydroxysulfide Nanosheets Electrocatalysts," *Adv Funct Mater*, vol. 30, no. 10, p. 1909610, Mar. 2020, doi: 10.1002/ADFM.201909610.

[26] B. You, N. Jiang, X. Liu, and Y. Sun, "Simultaneous H2 Generation and Biomass Upgrading in Water by an Efficient Noble-Metal-Free Bifunctional Electrocatalyst," *Angewandte Chemie International Edition*, vol. 55, no. 34, pp. 9913–9917, Aug. 2016, doi: 10.1002/ANIE.201603798.

[27] S. Sheng *et al.*, "Rational design of Co-S-P nanosheet arrays as bifunctional electrocatalysts for both ethanol oxidation reaction and hydrogen evolution reaction," *Inorg Chem Front*, vol. 7, no. 22, pp. 4498–4506, Nov. 2020, doi: 10.1039/D0QI00289E.

[28] J. Hao *et al.*, "In situ facile fabrication of Ni(OH)2 nanosheet arrays for electrocatalytic co-production of formate and hydrogen from methanol in alkaline solution," *Appl Catal B*, vol. 281, p. 119510, Feb. 2021, doi: 10.1016/J.APCATB.2020.119510.

[29] M. Li *et al.*, "Value-Added Formate Production from Selective Methanol Oxidation as Anodic Reaction to Enhance Electrochemical Hydrogen Cogeneration," *ChemSusChem*, vol. 13, no. 5, pp. 914–921, Mar. 2020, doi: 10.1002/CSSC.201902921.

[30] Y. Guo *et al.*, "Coupling Methanol Oxidation with Hydrogen Evolution on Bifunctional Co-Doped Rh Electrocatalyst for Efficient Hydrogen Generation," *Adv Funct Mater*, vol. 33, no. 2, p. 2209134, Jan. 2023, doi: 10.1002/ADFM.202209134.

[31] Y. Wu *et al.*, "Cyclic voltammetric deposition of binder-free Ni-Se film on Ni foams as efficient bifunctional electrocatalyst for boosting overall urea-water electrolysis," *J Alloys Compd*, vol. 937, p. 168460, Mar. 2023, doi: 10.1016/J.JALLCOM.2022.168460.

[32] D. Chanda, K. Kannan, J. Gautam, M.M. Meshesha, S.G. Jang, V.A. Dinh, B.L. Yang, Effect of the interfacial electronic coupling of nickel-iron sulfide nanosheets with layer Ti3C2 MXenes as efficient bifunctional electrocatalysts for anion-exchange membrane water electrolysis, Appl Catal B. 321 (2023) 122039. https://doi.org/10.1016/J.APCATB.2022.122039.

[33] Y. Zhao, Q. Wen, D. Huang, C. Jiao, Y. Liu, Y. Liu, J. Fang, M. Sun, L. Yu, Y. Zhao, M. Sun, L. Yu, Q. Wen, Y. Liu, D. Huang, J. Fang, C. Jiao, Operando Reconstruction toward Dual-Cation-Defects Co-Containing NiFe Oxyhydroxide for Ultralow Energy Consumption Industrial Water Splitting Electrolyzer, Adv Energy Mater. 13 (2023) 2203595. https://doi.org/10.1002/AENM.202203595.

[34] J. Sun, S. Qin, Z. Zhang, C. Li, X. Xu, Z. Li, X. Meng, Joule heating synthesis of well lattice-matched Co2Mo3O8/MoO2 heterointerfaces with greatly improved hydrogen evolution reaction in alkaline seawater electrolysis with 12.4 % STH efficiency, Appl Catal B. 338 (2023) 123015. https://doi.org/10.1016/J.APCATB.2023.123015.

[35] J. Sun, Z. Zhang, X. Meng, Low-Pt supported on MOF-derived Ni(OH)2 with highly-efficiently electrocatalytic seawater splitting at high current density, Appl Catal B. 331 (2023) 122703. https://doi.org/10.1016/J.APCATB.2023.122703.

[36] F.L. Wang, N. Xu, C.J. Yu, J.Y. Xie, B. Dong, X.Y. Zhang, Y.W. Dong, Y.L. Zhou, Y.M. Chai, Porous heterojunction of Ni2P/Ni7S6 with high crystalline phase and superior conductivity for industrial anion exchange membrane water electrolysis, Appl Catal B. 330 (2023) 122633. https://doi.org/10.1016/J.APCATB.2023.122633.

[37] H. Li, N. Yu, F. Gellrich, A.K. Reumert, M.R. Kraglund, J. Dong, D. Aili, J. Yang, Diamine crosslinked anion exchange membranes based on poly(vinyl benzyl methylpyrrolidinium) for alkaline water electrolysis, J Memb Sci. 633 (2021) 119418. https://doi.org/10.1016/J.MEMSCI.2021.119418.

[38] J.E. Park, M.J. Kim, M.S. Lim, S.Y. Kang, J.K. Kim, S.H. Oh, M. Her, Y.H. Cho, Y.E. Sung, Graphitic carbon nitride-carbon nanofiber as oxygen catalyst in anion-exchange membrane water electrolyzer and rechargeable metal–air cells, Appl Catal B. 237 (2018) 140–148. https://doi.org/10.1016/J.APCATB.2018.05.073.

[39] S.H. Ahn, B.S. Lee, I. Choi, S.J. Yoo, H.J. Kim, E.A. Cho, D. Henkensmeier, S.W. Nam, S.K. Kim, J.H. Jang, Development of a membrane electrode assembly for alkaline water electrolysis by direct electrodeposition of nickel on carbon papers, Appl Catal B. 154–155 (2014) 197–205. https://doi.org/10.1016/J.APCATB.2014.02.021.

[40] Y.S. Park, J. Jeong, Y. Noh, M.J. Jang, J. Lee, K.H. Lee, D.C. Lim, M.H. Seo, W.B. Kim, J. Yang,

S.M. Choi, Commercial anion exchange membrane water electrolyzer stack through non-precious metal electrocatalysts, Appl Catal B. 292 (2021) 120170. https://doi.org/10.1016/J.APCATB.2021.120170.
